# Importance of cholesterol-rich microdomains in the regulation of Nox isoforms and redox signaling in human vascular smooth muscle cells

**DOI:** 10.1038/s41598-020-73751-4

**Published:** 2020-10-20

**Authors:** Aikaterini Anagnostopoulou, Livia L. Camargo, Daniel Rodrigues, Augusto C. Montezano, Rhian M. Touyz

**Affiliations:** Institute of Cardiovascular and Medical Sciences, British Heart Foundation Glasgow Cardiovascular Research Centre, University of Glasgow, 126 University Place, Glasgow, G12 8TA UK

**Keywords:** Stress signalling, Caveolae

## Abstract

Vascular smooth muscle cell (VSMC) function is regulated by Nox-derived reactive oxygen species (ROS) and redox-dependent signaling in discrete cellular compartments. Whether cholesterol-rich microdomains (lipid rafts/caveolae) are involved in these processes is unclear. Here we examined the sub-cellular compartmentalization of Nox isoforms in lipid rafts/caveolae and assessed the role of these microdomains in VSMC ROS production and pro-contractile and growth signaling. Intact small arteries and primary VSMCs from humans were studied. Vessels from Cav-1^−/−^ mice were used to test proof of concept. Human VSMCs express Nox1, Nox4, Nox5 and Cav-1. Cell fractionation studies showed that Nox1 and Nox5 but not Nox4, localize in cholesterol-rich fractions in VSMCs. Angiotensin II (Ang II) stimulation induced trafficking into and out of lipid rafts/caveolae for Nox1 and Nox5 respectively. Co-immunoprecipitation studies showed interactions between Cav-1/Nox1 but not Cav-1/Nox5. Lipid raft/caveolae disruptors (methyl-β-cyclodextrin (MCD) and Nystatin) and Ang II stimulation variably increased O_2_^−^ generation and phosphorylation of MLC20, Ezrin-Radixin-Moesin (ERM) and p53 but not ERK1/2, effects recapitulated in Cav-1 silenced (siRNA) VSMCs. Nox inhibition prevented Ang II-induced phosphorylation of signaling molecules, specifically, ERK1/2 phosphorylation was attenuated by mellitin (Nox5 inhibitor) and Nox5 siRNA, while p53 phosphorylation was inhibited by NoxA1ds (Nox1 inhibitor). Ang II increased oxidation of DJ1, dual anti-oxidant and signaling molecule, through lipid raft/caveolae-dependent processes. Vessels from Cav-1^−/−^ mice exhibited increased O_2_^−^ generation and phosphorylation of ERM. We identify an important role for lipid rafts/caveolae that act as signaling platforms for Nox1 and Nox5 but not Nox4, in human VSMCs. Disruption of these microdomains promotes oxidative stress and Nox isoform-specific redox signalling important in vascular dysfunction associated with cardiovascular diseases.

## Introduction

Vascular smooth muscle cell (VSMC) function is regulated by vasoactive factors that signal through multiple membrane-associated receptors and downstream signalling molecules, including kinases, phosphatases, ion channels and transcription factors. Many of these processes are influenced by reactive oxygen species (ROS), particularly superoxide (O_2_^−^) and hydrogen peroxide (H_2_O_2_), and involve post-translational modification of signalling proteins, including oxidation and phosphorylation^1, 2^. Fundamental to these events is efficient orchestration and co-localization of signalling elements, which may involve plasma membrane cholesterol-rich microdomains that concentrate numerous receptors, G-proteins, and downstream molecules that function as effectors of extracellular signals^3, 4^.

Cholesterol-rich microdomains, enriched in cholesterol and sphingolipids, comprise lipid rafts and caveolae and are abundant in endothelial cells and VSMCs. Lipid rafts/caveolae act as platforms for several signalling pathways that control VSMC function. Caveolae are specialized sub-types of lipid rafts with a similar lipid profile but they also contain the scaffolding protein caveolin (Cav), of which there are 3 isoforms (Cav-1–3) (21–24 kDA)^5^. Of the Cav isoforms, Cav-1 is the most important for caveolae formation and function in VSMCs as demonstrated in Cav-1^−/−^ mice where VSMC caveolae are absent and caveolae-associated signalling is altered^6^. In particular cell proliferation is increased, eNOS is upregulated and vascular tone is impaired^7^. Cav-1 contains a scaffolding domain that interacts with several proteins in microdomains and regulates their activation or inhibition^8^. It also influences Ang II/ Ang II type 1 receptor (AT1R) signalling and trafficking, critically important in the regulation of VSMC function and vascular contraction^9, 10^.

In addition to acting as a platform for classical signalling pathways, growing evidence indicates that lipid rafts/caveolae play a regulatory role in ROS generation. Cav-1 knock-down or cholesterol depletion by methyl-β-cyclodextrin (MCD) in human lung fibroblasts and mouse macrophages leads to increased O_2_^−^ and H_2_O_2_ generation^11^. Cav-1 knockdown by siRNA in bovine aortic endothelial cells promotes increased mitochondrial ROS generation and in aortic endothelial cells from Cav-1^null^ mice H_2_O_2_ levels are increased^12^. Cav-1 silencing in VSMCs enhanced both basal and Ang II-induced mitochondrial ROS generation^13^. These phenomena are especially important in VSMCs, where redox-dependent signalling is involved in almost every functional vascular response. Molecular mechanisms regulating vascular redox signalling are complex because different types of ROS influence different downstream pathways. For example, O_2_^−^ is important in pro-contractile^14, 15^ and pro-inflammaory signalling^16^, whereas H_2_O_2_ promotes vasodilation^17, 18^. Processes controlling these differential responses may relate to sub-cellular compartmentalization of Noxs, the oxidases responsible for ROS generation in the vascular system.

Human vascular cells possess multiple Nox isoforms (Nox1, 2, 4, 5)^19, 20^, which have different functions in the vascular system: Nox1 promotes inflammation and proliferation^19, 20^, Nox2 induces inflammation, Nox4 is vasoprotective ^21, 22^ and Nox5 is pro-contractile^23, 24^. Nox1-4 require p22phox for their activation while Nox5 is a p22phox-independent isoform^19–24^ . Nox1, 2 and 5 are tightly regulated by vasoactive factors, whereas Nox4 is constitutively active^19–22^. Molecular mechanisms responsible for these variable processes are unclear, although compartmentalization in sub-cellular domains including lipid rafts has been suggested^3, 11^.

Other factors that modulate vascular ROS bioavailabity in VSMCs are antioxidant systems such as superoxide dismutase (SOD), catalase and peroxidases. In addition, PARK7 (Parkinson’s disease protein-7), also called DJ1, acts as an antioxidant and a signaling molecule. In its oxidized state, it activates nuclear factor erythroid-related factor 2 (Nrf2), a master regulator of anti-oxidant transcription factors^25, 26^. DJ1 localizes in the cytoplasm and in conditions of oxidative stress translocates to the mitochondria and nucleus^27–29^. It has also been shown to associate with Cav-1 in rat astrocytes and ventricular cardiomyocytes^30–32^. However it is unknown whether DJ1 associates with Noxs and Ang II signaling in VSMCs.

To better understand subcellular mechanisms that coordinate redox signaling we questioned the role of cholesterol-rich microdomains in Nox isoform regulation and ROS production and sought to evaluate whether these processes influence downstream redox-sensitive targets and pro-contractile and proliferative signaling in human VSMCs and vessels.

## Results

### Expression of Nox isoforms and localization with Cav-1 in human small arteries

**Figure 1:**
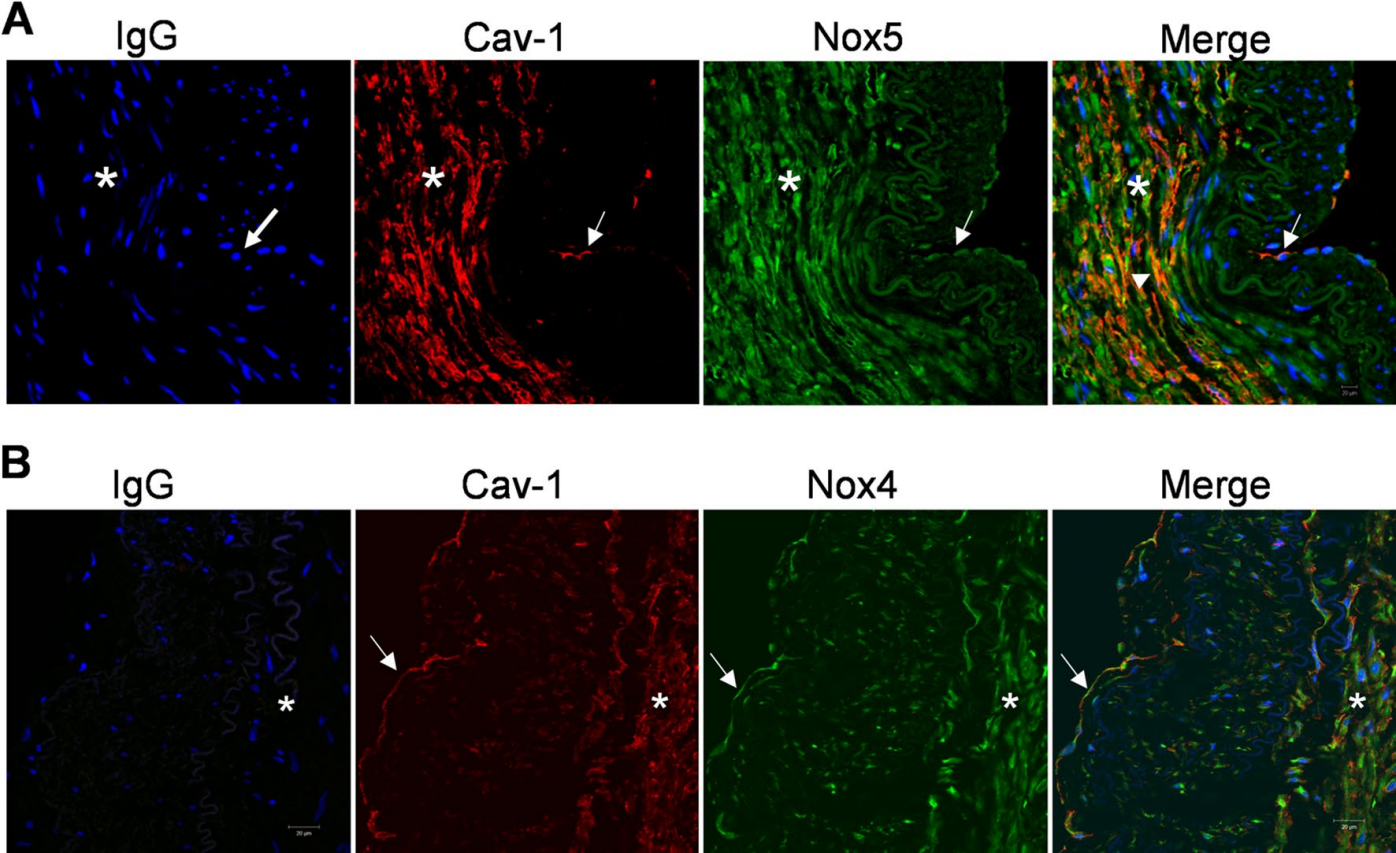
Localization of Nox isoforms and caveolin-1 (Cav-1) in humans small arteries. Paraffin-embedded sections of human small arteries were immunostained with Nox4, Nox5 and Cav-1. (**A**) Nox5 partially colocalizes with Cav-1 in the vascular media. (**B**) Nox4 does not collocalize with Cav-1. Green immunofluorescence indicates Nox4 or Nox5 immunostaining, red indicates Cav-1 staining and blue indicates nuclear staining using DAPI. After probing with the respective antibodies, slides were mounted, images were recorded in an Axiovert 200M microscope with a laser scanning module LSM 510 (Carl Zeiss AG, Heidelberg, Germany) using a × 40 objective. Image processing was obtained by Image J. Asterisk: vascular media comprising VSMCs. Arrows: Endothelium. Arrowhead: partial-colocalization of Nox5 and Cav-1 (orange colour). *Cav-1* Caveolin-1, *IgG* immunoglobulin G.

Cellular expression of Nox isoforms and co-localization with Cav-1 in intact human small arteries was assessed using double-labelling immunofluorescence. As shown in Fig. 1, Nox isoforms and Cav-1 are present in human arteries. Cav-1 localized mainly in the endothelium (intima) and vascular smooth muscle (media) layer. Nox5 was expressed both in the endothelium and vascular media, with greater abundance in the vascular smooth muscle layer. Nox4 was expressed both in the endothelium and vascular smooth muscle layer. As shown in Fig. 1A,B, there is a partial colocalization of Nox5 and Cav-1 as indicated by the orange colour. Nox4 does not seem to localize with Cav-1. We could not detect Nox1 in whole tissue in an isoform-specific manner by immunofluorescence despite multiple antibodies.

### Nox 1 and Nox5, but not Nox4, associate with cholesterol-rich microdomains in human VSMCs

**Figure 2:**
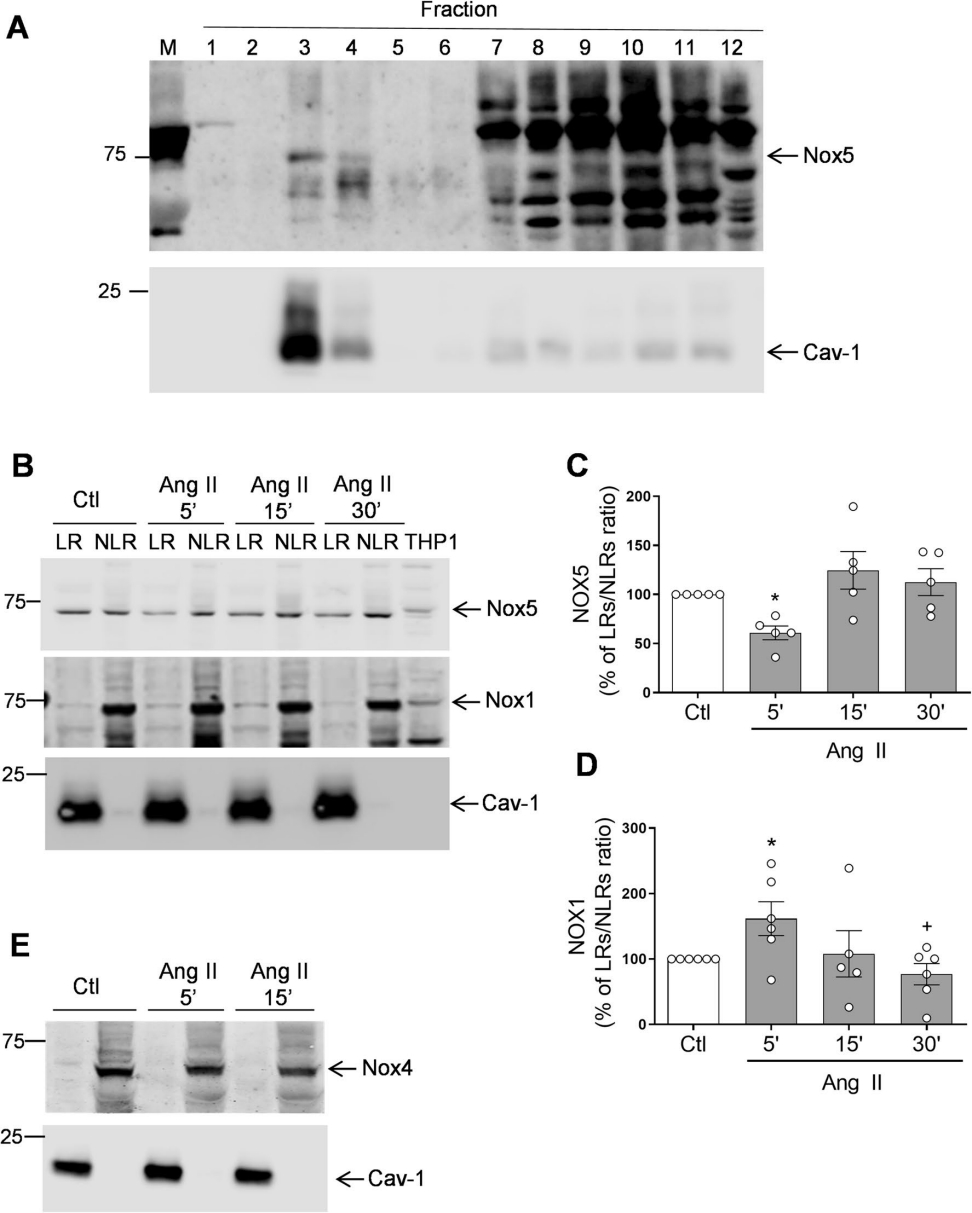
Nox1 and Nox5 but not Nox4 are present in lipid-rafts/caveolae in human VSMCs. VSMCs isolated from small arteries were stimulated with Ang II and subjected to discontinuous sucrose density gradient. (**A**) Representative immunoblots of Nox5 and Cav-1. Nox5 is present in isolated lipid-rafts fractions 3–4 in human VSMCs. (**B**) Representative images of Nox5 and Nox1 in lipid rafts (LRs) vs. non-lipid rafts (NLRs) in VSMCs stimulated with Ang II (100 nmol/L) for 5, 15 or 30 min. Isolated LR (fractions 3–4) or high density NLR (fractions 7–12) were pooled together and the percentage ratio of LRs vs. NLRs for Nox5 (**C**) and Nox1 (**D**) was determined. (**E**) Representative images of Nox4 in LRs vs. NLRs. Bar graphs are means ± SEM from 4–5 experiments. Control was taken as 100% and data are presented as the percentage changes relative to control conditions. *P < 0.05 vs. Ctl. ^#^P < 0.05 vs. Ang II, 5 min. *Ang II* angiotensin II, *Ctl* control, *Cav-1* caveolin-1, *LR* lipid rafts, *NLR* non-lipid rafts, *M* marker, *THP1* human acute monocytic leukemia cells, *VSMCs* vascular smooth muscle cells.

To evaluate in greater detail the sub-cellular localization of vascular Nox isoforms in cholesterol-rich microdomains and the impact of Ang II stimulation, we studied human VSMCs that were fractionated into cholesterol-rich and cholesterol-poor fractions using a detergent-free sucrose gradient centrifugation method. As shown in Fig. 2A, Nox5 was expressed in fractions 3–4, corresponding to cholesterol-rich fractions and which likely comprise lipid rafts/caveolae as confirmed by abundant Cav-1 expression. Nox5 was also present in the high density non-lipid raft fractions (fractions 7–12), possibly reflecting cytoplasmic localization. In subsequent studies, we combined lipid rich (fractions 3, 4) and non lipid-rich (fractions 7–12) fractions and showed that while Nox1 and Nox5 are expressed in both lipid-raft and high density non-lipid rafts fractions, abundance of Nox1 and Nox5 was greater in the high density versus the the low density fractions (Fig. 2B–D). Nox4 was abundantly expressed in high density non-lipid fractions and absent in low density fractions, suggesting that Nox4, while expressed in VSMCs, does not localize in cholesterol-rich microdomains (Fig. 2E). These findings support those of others who also failed to show Nox4 in lipid rafts in VSMCs and endothelial cells^3, 11^.

### Ang II stimulates lipid raft/caveoale trafficking of Nox1 and Nox5 but not Nox1

As shown in Fig. 2B,C, Ang II stimulation resulted in rapid translocation of Nox5 (within 5 min) out of the lipid-raft fractions into the high density non-lipid raft fractions. On the other hand, Ang II stimulation induced Nox1 trafficking into lipid raft fractions (Fig. 2D). Ang II did not have any effect on Nox4 lipid-raft trafficking.

### Cholesterol-rich microdomains negatively regulate Nox-derived superoxide production in human VSMC

**Figure 3:**
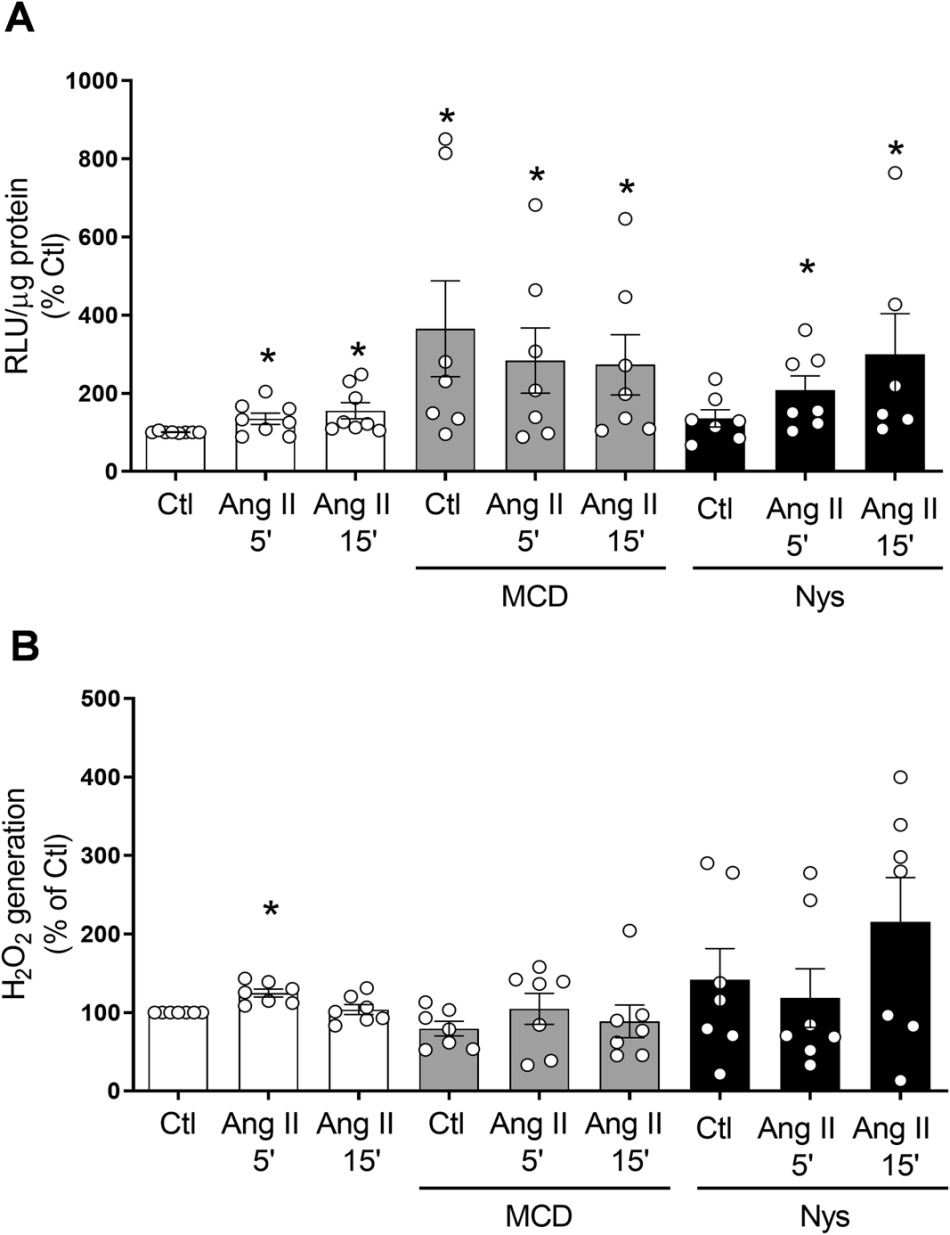
Differential effects of lipid-raft disruption on Ang II–induced ROS production. VSMCs were treated with methyl-b-cyclodextrin (MCD; 10 mmol/L) for 45 min or Nystatin (Nys; 50 μg/mL) for 30 min and stimulated with Ang II (100 nmol/L) for 5 and 15 min. (**A**) NADPH-derived O_2_^−^ generation measured by lucigenin assay in human VSMCs. (**B**) H_2_O_2_ levels were measured by the Amplex Red assay. Data are mean ± SEM from 5–12 experiments. Control was taken as 100% and data are presented as the percentage changes relative to control conditions. *P < 0.05 vs. Ctl. *Ang II* angiotensin II, *Ctl* control, *H*_*2*_*O*_*2*_ hydrogen peroxide, *RLU* relative light units.

To examine the functional significance of lipid rafts/caveolae in Nox-derived ROS production in basal and Ang II-stimulated VSMCs, we examined generation of O_2_^−^ and H_2_O_2_ in Ang II-stimulated cells treated with or without MCD or nystatin. In basal conditions VSMCs exposed to MCD exhibited significantly increased NADPH-derived O_2_^−^ levels compared with vehicle-treated cells in which lipid rafts were intact (Fig. 3A). Ang II significantly increased NADPH-derived O_2_^−^ production compared with basal conditions. Ang II increased O_2_^−^ production in Nys-treated VSMCs (Fig. 3A). Lipid-raft disruption by MCD or Nys did not have an effect on H_2_O_2_ levels in human VSMCs (Fig. 3B). Together these results suggest that Noxs that generate primarily O_2_^−^, including Nox1 and Nox5, are likley more tightly regulated by lipid rafts/caveolae than Noxs that generate mainly H_2_O_2_, specifically Nox4.

### Ang II-induced generation of O_2_^−^ and H_2_O_2_ in human VSMCs involves different Nox isoforms

**Figure 4:**
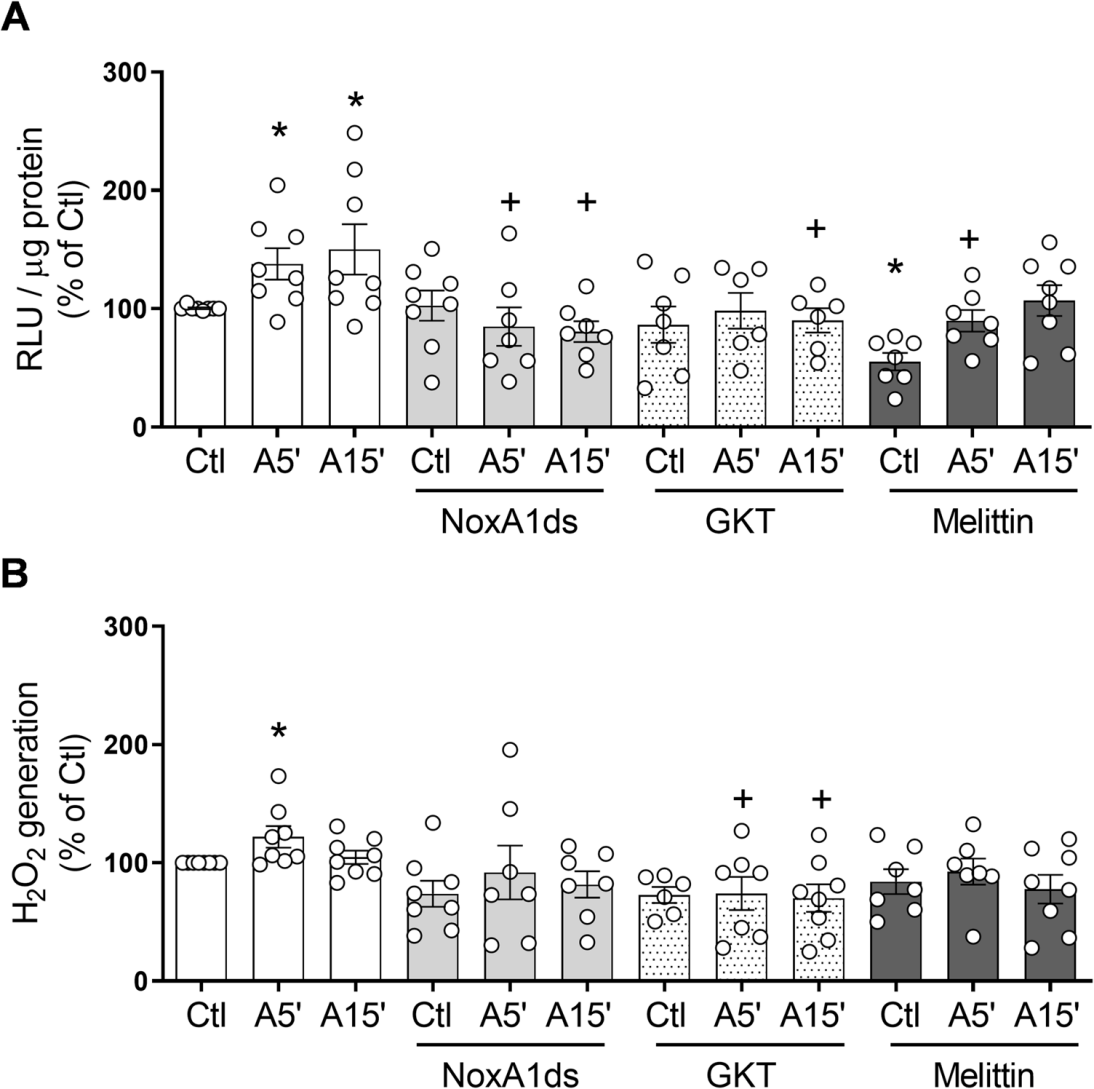
Nox isoforms differentially regulate Ang II-induced ROS production in human VSMC. Cells were stimulated with Ang II (100 nmol/L) for 5 and 15 min in the presence and absence of NoxA1ds (Nox1 inhibitor, 10 μmol/L), GKT137831 (Nox1/Nox4 inhibitor, 10 μmol/L) and melittin (Nox5 inhibitor, 100 nmol/L). NADPH dependent O_2_^−^ production (**A**) and H_2_O_2_ levels (**B**) were measured by lucigenin-derived chemiluminescence and amplex red, respectively. Results are expressed as mean ± SEM of 6–8 separate experiments. *P < 0.05 vs. Ctl, ^+^P < 0.05 vs. respective Ang II group. *Ang II* angiotensin II, *Ctl* control, *H*_*2*_*O*_*2*_ hydrogen peroxide, *RLU* relative light units.

NADPH-dependent O_2_^−^ generation and H_2_O_2_ levels were assessed in Ang II-stimulated VSMCs in the absence and presence of Nox isoform inhibitors. As shown in Fig. 4 Ang II induced an increase in NADPH-dependent O_2_^−^ generation and H_2_O_2_ within 5 min of stimulation. NoxA1ds, GKT137831 and mellitin reduced Ang II induced O_2_^−^ generation at variable time points (Fig. 4A). Ang II-stimulated increase in H_2_O_2_ levels was reduced only by the Nox4 inhibitor GKT137831 (Fig. 4B). These data further support our findings that Nox1 and Nox5, which localise in cholesterol-rich domains, play an important role in Ang II-stimulated O_2_^−^ production, whereas Nox4, localised mainly in the cytoplasmic milieu, regulates H_2_O_2_ generation in response to Ang II.

### Disruption of cholesterol-rich microdomains upregulates vascular redox signalling

**Figure 5:**
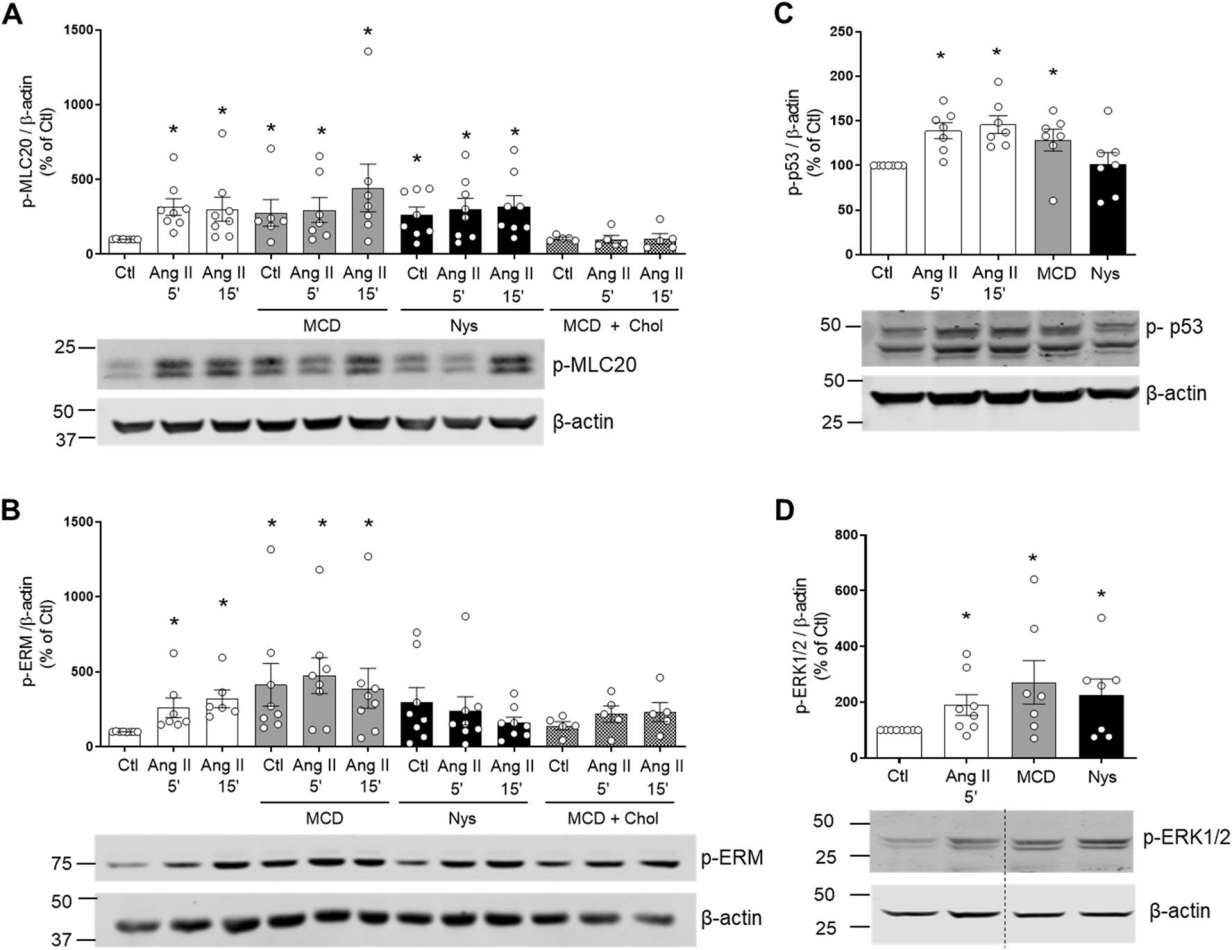
Effect of lipid-raft disrupters on phosphorylation of MLC20, Ezrin/Radixin/Moesin, p53 and ERK1/2 in Ang II-stimulated human VSMCs. VSMCs were stimulated with Ang II (100 nmol/L) for 5 and 15 min in the presence and absence of MCD (10 mmol/L) or Nys (50 μg/mL). In some experiments, after MCD treatment, cholesterol was reloaded with cholesterol:MCD (1–10 mmol/L) complex. Phosphorylation of MLC20 (**A**), Ezrin–Radixin–Moesin (**B**), p53 (**C**) and ERK1/2 (**D**) were analysed by western blot. β-Actin was used as loading control. Bar graphs are means ± SEM from 5–7 experiments. Control was taken as 100% and data are presented as the percentage changes relative to control conditions. *P < 0.05 vs. Ctl. *Ang II* angiotensin II, *Ctl* control, *MCD* methyl-b-cyclodextrin, *Nys* nystatin, *Chol* cholesterol, *p-MLC20* phosphorylated MLC20, *p-ERM* phosphorylated Ezrin–Radixin–Moesin, *p-p53* phosphorylated p53, *p-ERK1/2* phosphorylated ERK1/2.

To investigate whether cholesterol-rich microdomains influence redox-sensitive signal transduction in VSMCs, we examined signalling molecules involved in contraction (MLC20), actin cytoskeletal organization (Ezrin–Radixin–Moesin), apoptosis/cell cycle (p53) and growth (ERK1/2) in Ang II-stimulated VSMCs in the absence and presence of MCD and nystatin. As shown in Fig. 5, within 5 min of stimulation, Ang II significantly increased phosphorylation of MLC20 (Ang II 5′: 275.0 ± 47.04% vs. Veh; Ang II 15′: 363.7 ± 121.2% vs. Veh; p < 0.05, Fig. 5A), Ezrin-Radixin-Moesin (Ang II 5′: 346.1 ± 119.0% vs. Veh; Ang II 15′: 260.1 ± 67.82% vs. Veh; p < 0.05, Fig. 5B), p53 (Ang II 5′: 133.5 ± 8.210% vs. Veh; Ang II 15′:149.9 ± 10.68% vs. Veh; p < 0.05, Fig. 5C) and ERK1/2 (Ang II 5′: 189.6 ± 37.23% vs. Veh; p < 0.05, Fig. 5D). Lipid raft disruptors, MCD and nystatin, increased expression of phospho-MLC20 (MCD: 329.9 ± 97.77% vs. Veh; MCD, Ang II 5′:341.5 ± 109.6% vs. Veh; Nys: 257.8 ± 75.89%; Nys, Ang II 15′: 372.3 ± 111.1% vs. Veh; p < 0.05, Fig. 5A), phospho-Ezrin-Radixin-Moesin (MCD, Ang II 5′: 474.5 ± 137.5, p < 0.05, Fig. 5B) and phospho-p53 (MCD: 128.4 ± 12.25% vs. Veh; p < 0.05, Fig. 5C). No additional effects of Ang II were observed. Cholesterol reloading prevented phosphorylation of MLC20 and Ezrin–Radisin–Moesin in MCD-treated cells.

### Nox isoforms differentially regulate Ang II signaling

**Figure 6:**
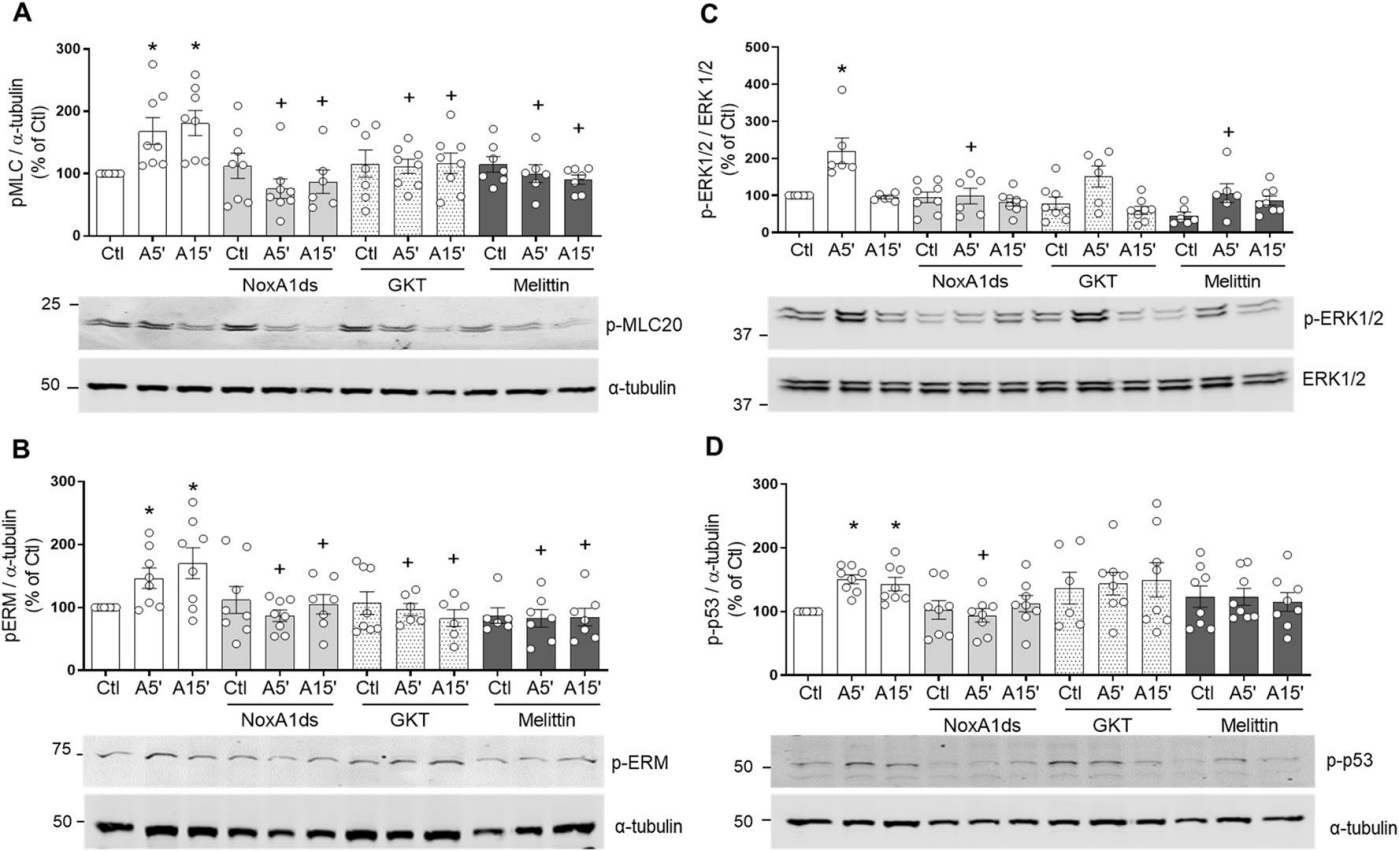
Nox1, Nox4 and Nox5 are involved in Ang II-induced redox signalling in human VSMC. Cells were stimulated with Ang II, (100 nmol/L) for 5 and 15 min in the presence and absence of NoxA1ds (Nox1 inhibitor, 10 μmol/L), GKT137831 (Nox1 and Nox4 inhibitor, 10 μmol/L) and melittin (Nox5 inhibitor, 10 nmol/L). Phosphorylation of MLC (**A**), Ezrin-Radixin-Moesin (**B**), p53 (**C**) and ERK1/2 (**D**) was detected by western blotting in hVSMC. α-Tubulin and total ERK1/2 were used as loading control. Results are expressed as mean ± SEM of 6–8 separate experiments. *P < 0.05 vs. control (Ctl), ^+^P < 0.05 vs. respective Ang II group. *Ang II* angiotensin II, *Ctl* control, *p-MLC20* phosphorylated MLC20, *p-ERM* phosphorylated Ezrin–Radixin–Moesin, *p-p53* phosphorylated p53, *p-ERK1/2* phosphorylated ERK1/2.

We used two approaches to interrogate the role of Nox isoforms in Ang II signaling, (1) pharmacological inhibitors and (2) siRNA targeting. Cells were pretreated with Nox isoform-specific pharmacological inhibitors including NoxA1ds (Nox1 inhibitor), GKT137831 (Nox1/4 inhibitor) and melittin (Nox5 inhibitor). These inhibitors have been extensively used and well characterised^33–35^. As shown in Fig. 6 after 5 min of stimulation, Ang II significantly increased phosphorylation of MLC20, Ezrin-Radixin-Moesin, ERK1/2 and, p53. GKT137831 and mellitin significantly reduced Ang II-induced phosphorylation of MLC (Fig. 6A) and Ezrin-Radixin-Moesin (Fig. 6B). NoxA1ds and mellitin significantly inhibited ERK1/2 phosphorylation (Fig. 6C) while p53 phosphorylation was attenuated only by NoxA1ds (Fig. 6D).

**Figure 7:**
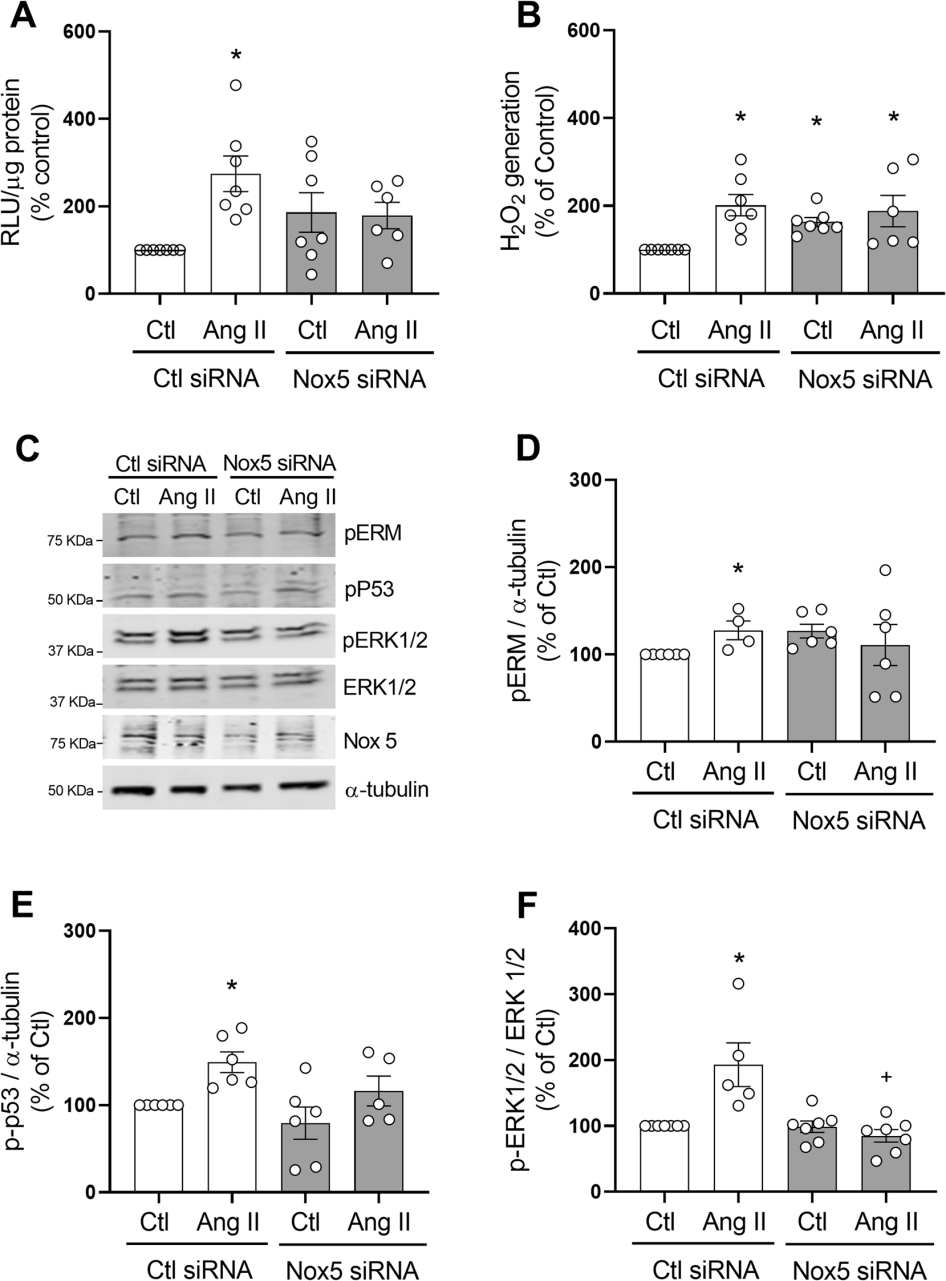
Effect of Nox5 silencing on ROS production and Ang II-induced signaling in hVSMC. Cells were transfected with Nox5 or control (Ctl) siRNA and stimulated with Ang II (100 nmol/L) for 5 min. NADPH-derived O_2_^−^ generation was measured by lucigenin assay (**A**). H_2_O_2_ levels were measured by the Amplex Red assay (**B**). Representaive images of phospho-Ezrin–Radixin–Moesin, phospho-p53, phospho-ERK1/2 and Nox5 detected by western blot. α-tubulin and total ERK1/2 were used as loading control (**C**). Protein quantification of Ezrin–Radixin–Moesin (**D**), p53 (**E**) and ERK1/2 (**F**) phosphorylation in hVSMCs. Data are means ± SEM from 4–7 experiments. Control was taken as 100% and data are presented as the percentage changes relative to control conditions. *P < 0.05 vs. control, ^+^P < 0.05 vs. AngII Ctl siRNA. *Ang II* angiotensin II, *Ctl* control, *H*_*2*_*O*_*2*_ hydrogen peroxide, *RLU* relative light units, *p-MLC20* phosphorylated MLC20, *p-ERM* phosphorylated Ezrin–Radixin–Moesin, *p-p53* phosphorylated p53, *p-ERK1/2* phosphorylated ERK1/2.

To specifically investigate the effects of Nox5 on Ang II-induced ROS production and redox signaling, cells were transfected with Nox5 siRNA (Supplementary figure 1A). In basal conditions, Nox5 silencing did not significantly alter O_2_^−^ levels (Fig. 7A), but increased H_2_O_2_ levels (Fig. 7B, Nox5 siRNA 63.3 ± 27.69% increase vs. Control, p < 0.05) compared to control siRNA. In Nox5 downregulated cells, Ang II failed to increase O_2_^−^ and had no effect on H_2_O_2_ production.

Nox5 silencing differentially influenced Ang II signaling in VSMC as shown in Fig. 7C–F. Nox5 downregulation did not alter phosphorylation of Ezrin/Radixin/Moesin, p-53 and ERK1/2 in basal conditions. In control siRNA cells, Ang II stimulation induced a significant increase in phosphorylation of Ezrin/Radixin/Moesin, p-53 and ERK1/2 compared with control counterparts. In Nox5 siRNA-treated cells, Ang II failed to increase phosphorylation of target proteins compared with control counterparts. Ang II-induced activation of ERK1/2 was significantly lower in Nox5 siRNA cells versus control siRNA cells (Nox5 siRNA 89.5 ± 9.56% increase vs. Control 193 ± 33.2%, p < 0.05).

**Figure 8:**
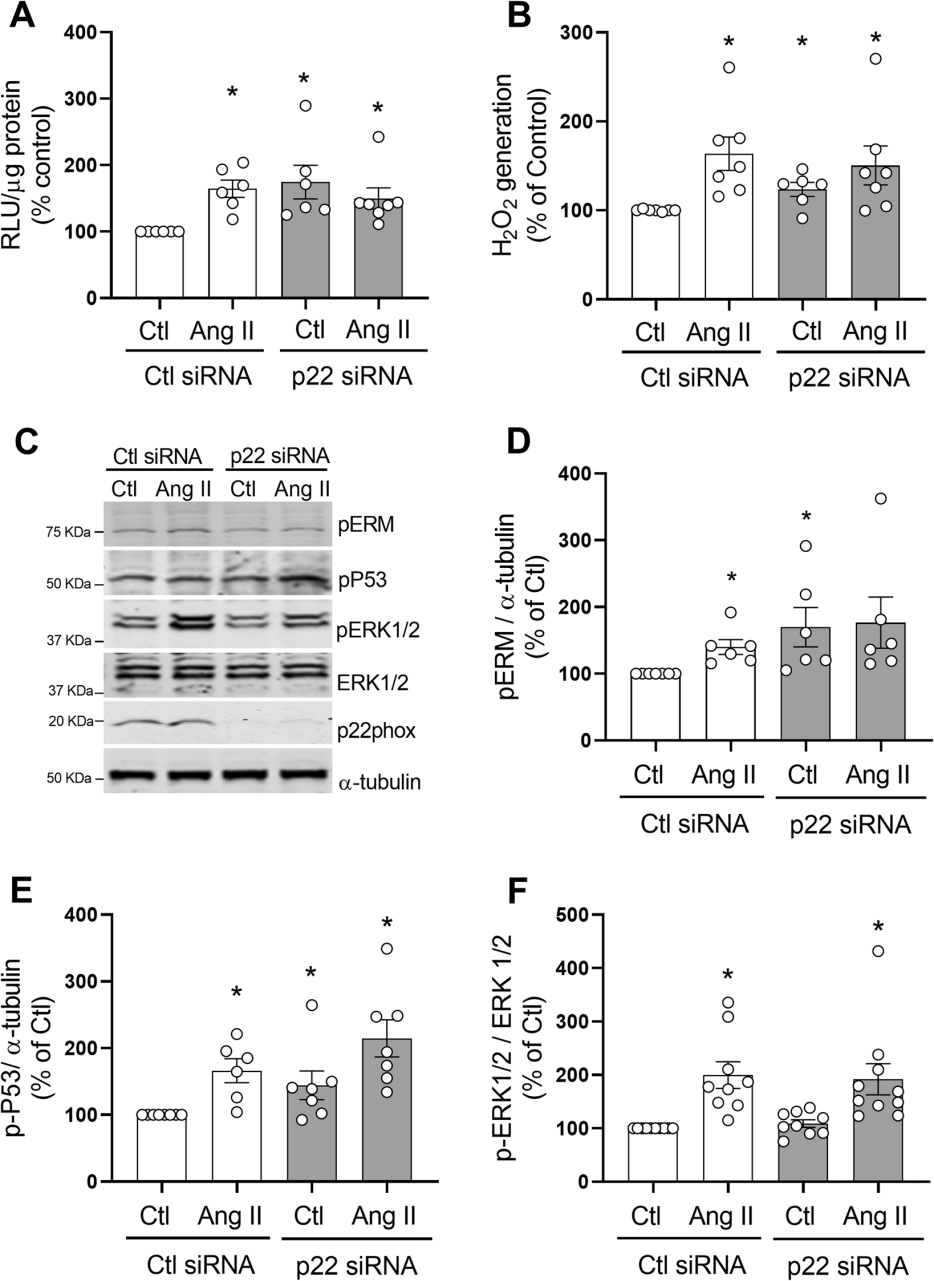
p22phox silencing effect on ROS production and Ang II-induced signaling in hVSMC. Cells were transfected with p22phox or control (Ctl) siRNA and stimulated with Ang II (100 nmol/L) for 5 min. NADPH-derived O_2_^−^ generation was measured by lucigenin assay (**A**). H_2_O_2_ levels were measured by the Amplex Red assay (**B**). Representaive images of phospho-Ezrin–Radixin–Moesin, phospho-p53, phospho-ERK1/2 and p22phox detected by western blot. α-Tubulin and total ERK1/2 were used as loading control (**C**). Protein quantification of Ezrin–Radixin–Moesin (**D**), p53 (**E**) and ERK1/2 (**F**) phosphorylation in hVSMCs. Data are means ± SEM from 4–7 experiments. Control was taken as 100% and data are presented as the percentage changes relative to control conditions. *P < 0.05 vs. control, ^+^P < 0.05 vs. AngII Ctl siRNA. *Ang II* angiotensin II, *Ctl* control, *H*_*2*_*O*_*2*_ hydrogen peroxide, *RLU* relative light units, *p-MLC20* phosphorylated MLC20, *p-ERM* phosphorylated Ezrin–Radixin–Moesin, *p-p53* phosphorylated p53, *p-ERK1/2* phosphorylated ERK1/2.

To investigate the role of of Nox1–4 (p22phox-dependent Nox isoforms) in Ang II-induced signaling, p22phox was silenced in VSMCs using siRNA (Supplementary figure 2A). In basal conditions, p22phox silencing increased production of O_2_^−^ (Fig. 8A, p22phox siRNA 63.3 ± 27.69% increase vs. Control, p < 0.05) and H_2_O_2_ in hVSMC (Fig. 8B, p22phox siRNA 23.5 ± 7.84% increase vs. Control, p < 0.05). Ang II did not cause a further increase in O_2_^−^ or H_2_O_2_ levels in cells transfected with p22phox siRNA.

Silencing of p22phox influenced Ang II signaling in VSMCs as shown in Fig. 8C–F. In p22phox siRNA-treated cells, basal levels of phosphorylated Ezrin/Radixin/Moesin (p22phox siRNA 69.7 ± 29.57% increase vs. Control, p < 0.05) and p53 (p22phox siRNA 44.7 ± 21.54% increase vs. Control, p < 0.05) were increased. In contrast, basal and Ang II-induced ERK1/2 phosphorylation was not affected by p22phox silencing compared to control siRNA.

### Ang II influences redox regulation of DJ1 in human VSMCs

To investigate whether other redox-sensitive targets important in vascular cell function are influenced by the lipid rafts/caveolae-Nox system, we investgated effects on DJ1, a dual antioxidant and signaling molecule, that we found to be abundantly expressed in human small arteries (Supplementary figure 2) and which interacts with Cav-1 and Nox1 (Supplementary figure 3). As shown in Supplementary figures 3A–2C, DJ1 physically associated with Nox1 but not with Nox5. Interactions between DJ1 and Nox1 were also observed by immunofluorescence, where Nox1 and DJ1 co-localized in VSMCs as shown in Supplemental figure 2D by the yellow fluorescence in merged images.

**Figure 9:**
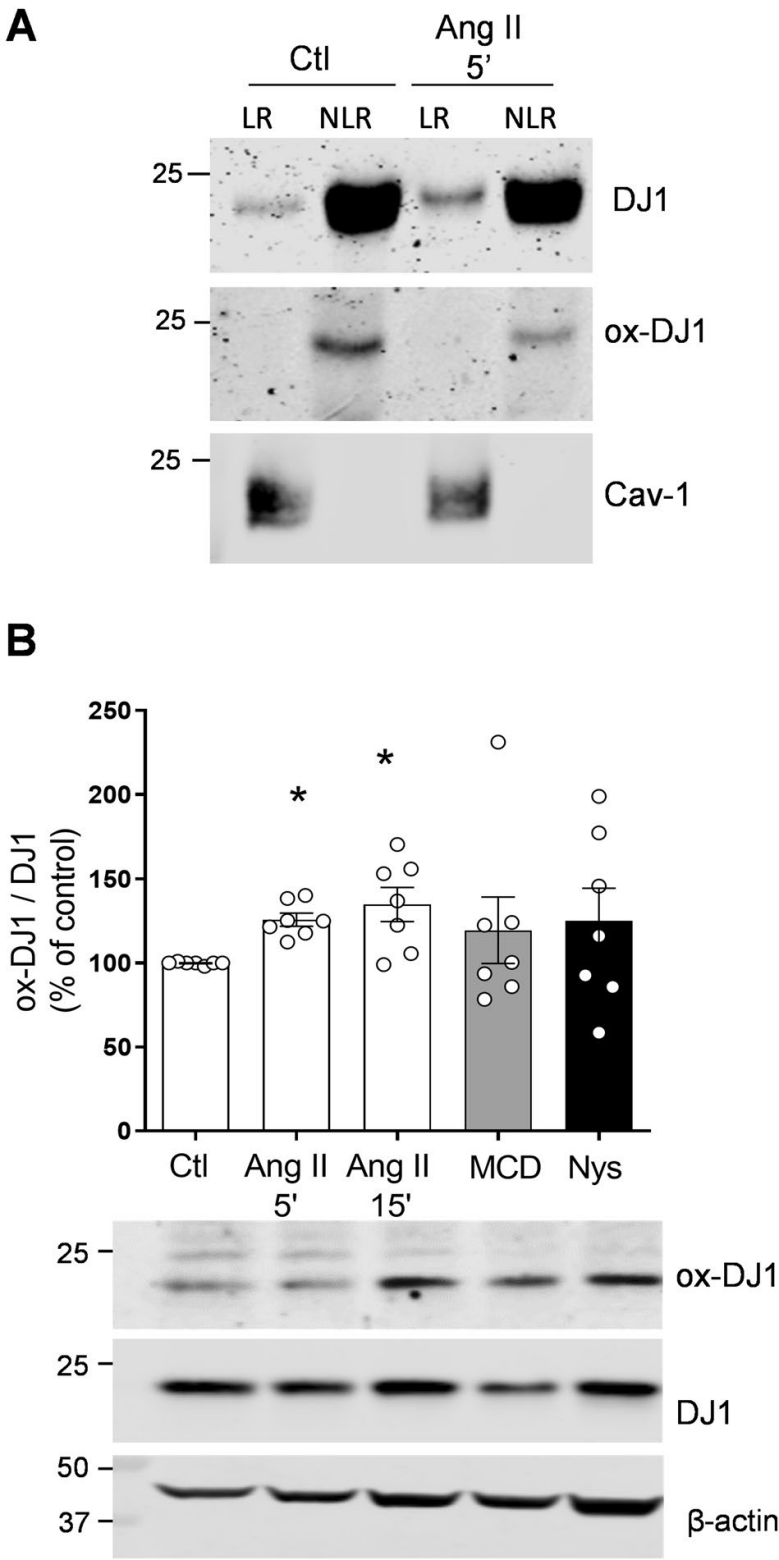
DJ1 oxidation—effects of Ang II and lipid raft disruptors. VSMCs treated with Ang II were subjected to discontinuous sucrose density gradient. (**A**) Representative immunoblots of total and irreversibly oxidised DJ1 in lipid rafts (LRs) vs. non-lipid rafts (NLRs). (**B**) VSMCs were stimulated with Ang II (100 nmol/L, 5 and 15 min) or treated with lipid-raft disruptors (10 mmol/L MCD or 50 μg/mL Nys). Representative immunoblots and quantification of total and irreversible oxidised DJ1. β-Actin was used as loading control. Data are means ± SEM from 7 experiments. Control was taken as 100% and data are presented as the percentage changes relative to control conditions. *P < 0.05 vs. control. *Ang II* angiotensin II, *Ctl* control, *Cav-1* caveolin-1, *LR* lipid raft, *NLR* non-lipid raft, *M* marker, *MCD* methyl-b-cyclodextrin, *Nys* nystatin.

Having demonstrated that human vessels and VSMCs abundantly express DJ1, we questioned whether Ang II regulates redox-sensitive (oxidized) DJ1 through prcesses involving cholesterol-rich microdomains. Human VSMCs were probed with specific antibodies against total and irreversibly oxidised DJ1 (Cys 106-SO_2_H; Cys 106-SO_3_H). DJ1 expression was examined in isolated lipid-rafts and non-lipid raft fractions of VSMCs stimulated with or without Ang II (10^−7^ M) for 5 min. As shown in Fig. 9A, the irreversibly oxidised form of DJ1 was only present in non-lipid raft fractions even though total DJ1 was present in lipid-rafts. These findings indicate that either non-oxidised or only reversibly oxidised DJ1 is present in rafts. Ang II increased irreversible oxidation of DJ1 within 5 min (Ang II 5′: 125.8 ± 3.870% vs. Veh, Ang II 15′: 134.8 ± 10.17% vs. Veh; p < 0.005) as shown in Fig. 9B. Lipid-raft disruption by MCD or nystatin induced an increase of irreversible oxidation of DJ1, a trend that did not reach statistical significance. The irreversibly oxidized form of DJ1 represents increased activation of DJ1 and identifies a novel downstream signaling target of Ang II.

### Cav-1 silencing increases ROS production and redox signaling in hVSMC

**Figure 10:**
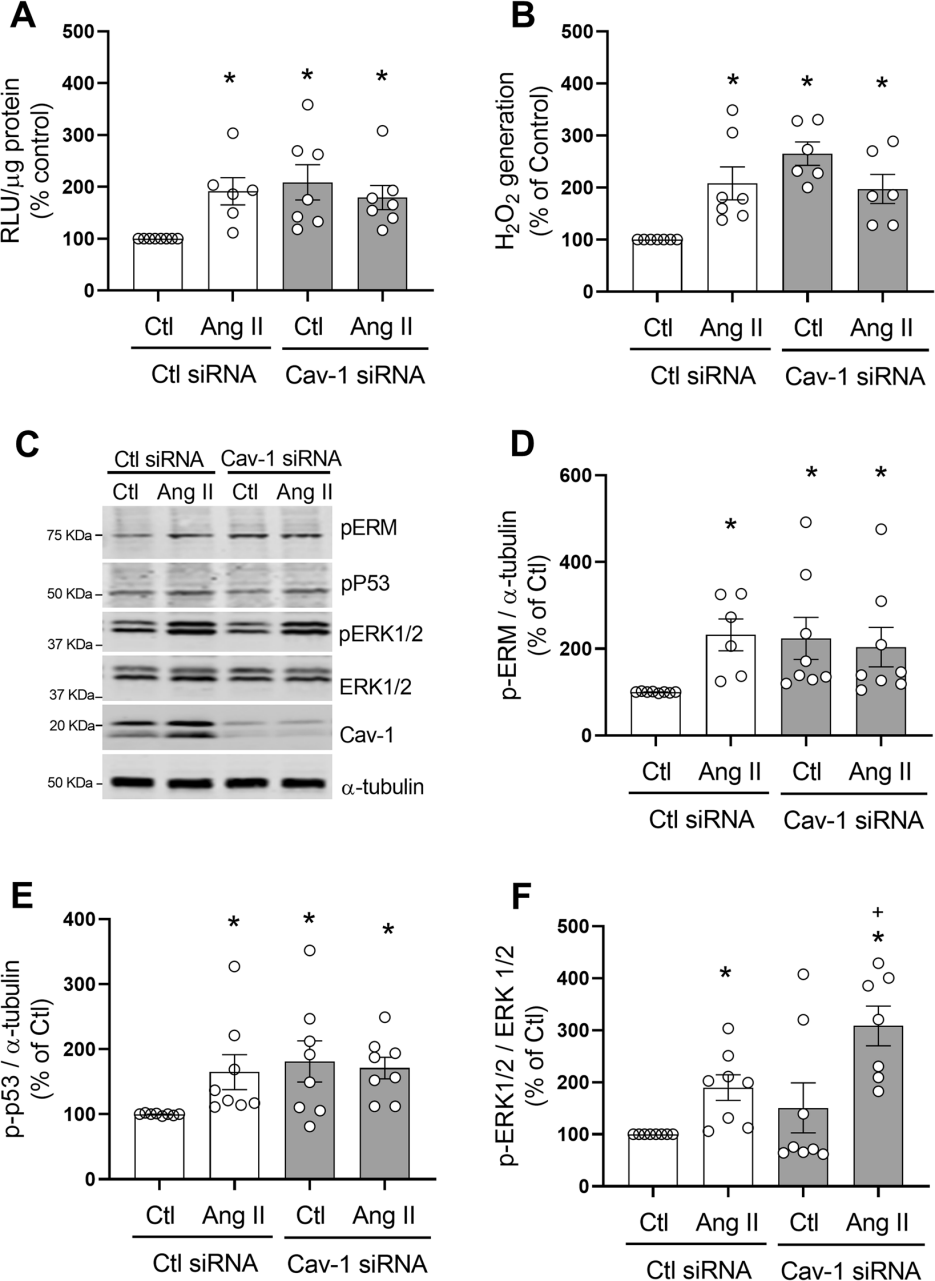
Cav-1 silencing increases ROS production and redox signaling in hVSMC. Cells were transfected with Cav-1 or control (Ctl) siRNA and stimulated with Ang II (100 nmol/L) for 5 min. NADPH-derived O_2_^−^ generation measured by lucigenin assay (**A**). H_2_O_2_ levels were measured by the Amplex Red assay (**B**). Representaive images of phospho-Ezrin–Radixin–Moesin, phospho-p53, phospho-ERK1/2 and Cav-1 detected by western blot. α-tubulin and total ERK1/2 were used as loading control (**C**). Protein quantification of Ezrin–Radixin–Moesin (**D**), p53 (**E**) and ERK1/2 (**F**) phosphorylation in hVSMCs. Data are means ± SEM from 6–8 experiments. Control was taken as 100% and data are presented as the percentage changes relative to control conditions. *P < 0.05 vs. control, ^+^P < 0.05 vs. AngII Ctl siRNA. *Cav-1* caveolin-1, *Ang II* angiotensin II, *Ctl* control, *H*_*2*_*O*_*2*_ hydrogen peroxide, *RLU* relative light units, *p-MLC20* phosphorylated MLC20, *p-ERM* phosphorylated Ezrin–Radixin–Moesin, *p-p53* phosphorylated p53, *p-ERK1/2* phosphorylated ERK1/2.

To further investigate the relationship between lipid rafts/caveolae, ROS and Ang II-induced signaling, we investigated effects of Cav-1 silencing (with siRNA; Supplementary figure 4) on ROS generation and Ang II-mediated redox signaling in hVSMC. As shown in Fig. 10A, in basal conditions, Cav-1 silencing resulted in an increase in NADPH-dependent O_2_^−^ levels compared to control siRNA (Cav-1siRNA 108.06 ± 33.98% increase vs. Control, p < 0.05). A similar response was observed for H_2_O_2_ (Fig. 10B), since cells transfected with Cav-1 siRNA showed higher H_2_O_2_ levels compared to control (Cav-1siRNA 165.3 ± 22.39% increase vs. Control, p < 0.05). Ang II did not cause a further increase in NADPH-dependent O_2_^−^ or H_2_O_2_ levels in Cav-1 siRNA-treated cells.

In addition to impacting ROS production, Cav-1 silencing influenced signaling in hVSMCs as shown in Fig. 10C–F. Cav-1 downregulation resulted in an increase in basal levels of phospho-Ezrin/Radixin/Moesin and phospho-p53 but not ERK1/2. In Ang II-stimulated cells, phosphorylation of ERK1/2, but not Ezrin/Radixin/Moesin or phospho-p53, was increased.

### ROS generation and redox-sensitive signalling are increased in arteries from caveolin-1^−/−^ mice

**Figure 11:**
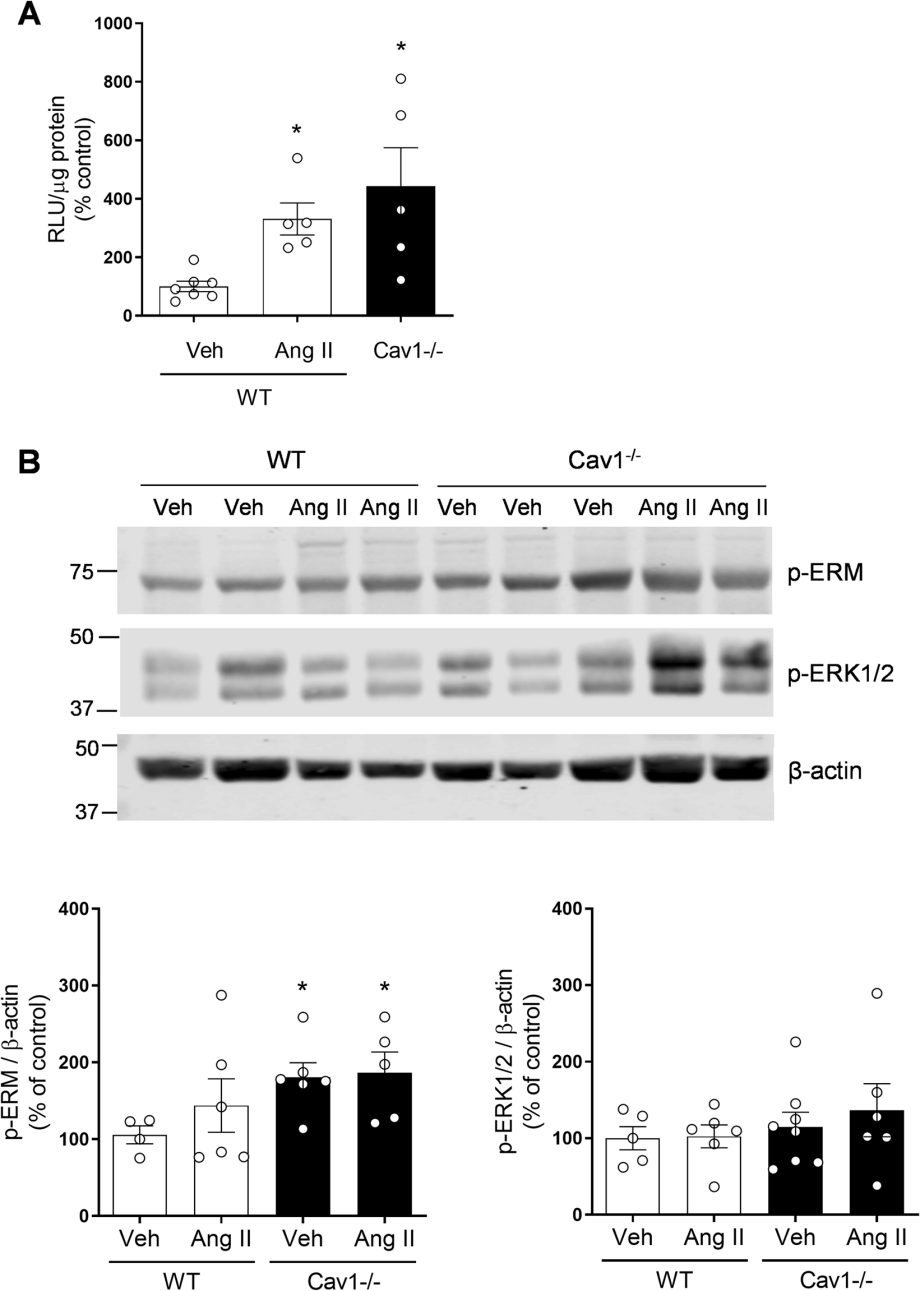
ROS generation and vascular signaling in Cav-1-deficient mice. Wild type (WT) and Cav-1^−/−^ mice were infused with vehicle (Veh; saline) or Ang II (400 ng/kg/min) and after two weeks, mesenteric arteries and aortas were isolated for NADPH-dependent ROS generation measurement and protein expression. (**A**) NADPH-derived O_2_^−^ generation measured by lucigenin assay in mesenteric arteries. (**B**) Phosphorylation of Ezrin/Radixin/Moesin and ERK1/2 in aortas isolated from WT and Cav-1^−/−^ mice. β-Actin was used as loading control. Data are means ± SEM from 5–7 experiments. *P < 0.05 vs. WT. *Ang II* angiotensin II, *Cav-1* caveolin-1, *Cav-1*^*−/−*^ caveolin-1 deficiency, *Veh* vehicle, *RLU* relative light units, *p-ERM* phosphorylated Ezrin–Radixin–Moesin, *p-ERK1/2* phosphorylated ERK1/2.

To test proof of concept from our human studies, we investigated whether our in vitro findings in human VSMCs are recapitulated in intact arteries in vivo. We studied isolated arteries from wild-type, and Cav-1^−/−^ mice and probed for Nox-dependent O_2_^−^ production and activation of redox-sensitive signalling pathways, ERK1/2 and Ezrin-Radixin-moisin. As shown in Fig. 11A, mesenteric arteries isolated from Cav-1^−/−^ mice had significantly higher levels of NADPH-dependent O_2_^−^ levels compared to wild-type mice (Cav-1^−/−^, 443.02 ± 131.70% increase vs. Control, p < 0.05). Aortas isolated from Cav-1^−/−^ mice had significantly greater expression of phospho-Ezrin/Radixin/Moesin compared with control mice while ERK1/2 phosphorylation was not significantly altered between groups (Fig. 11B). These findings suggest that in the absence of regulated caveolae (Cav-1^−/−^), Nox-dependent ROS production and activation of signalling pathways associated with VSMC actin cytoskeletal organisation are increased, indicating an important role for intact lipid rafts/caveolae (Cav-1) in the regulation of Nox activity, ROS generation and vascular signalling, findings recapitulated in human VSMCs.

## Discussion

**Figure 12:**
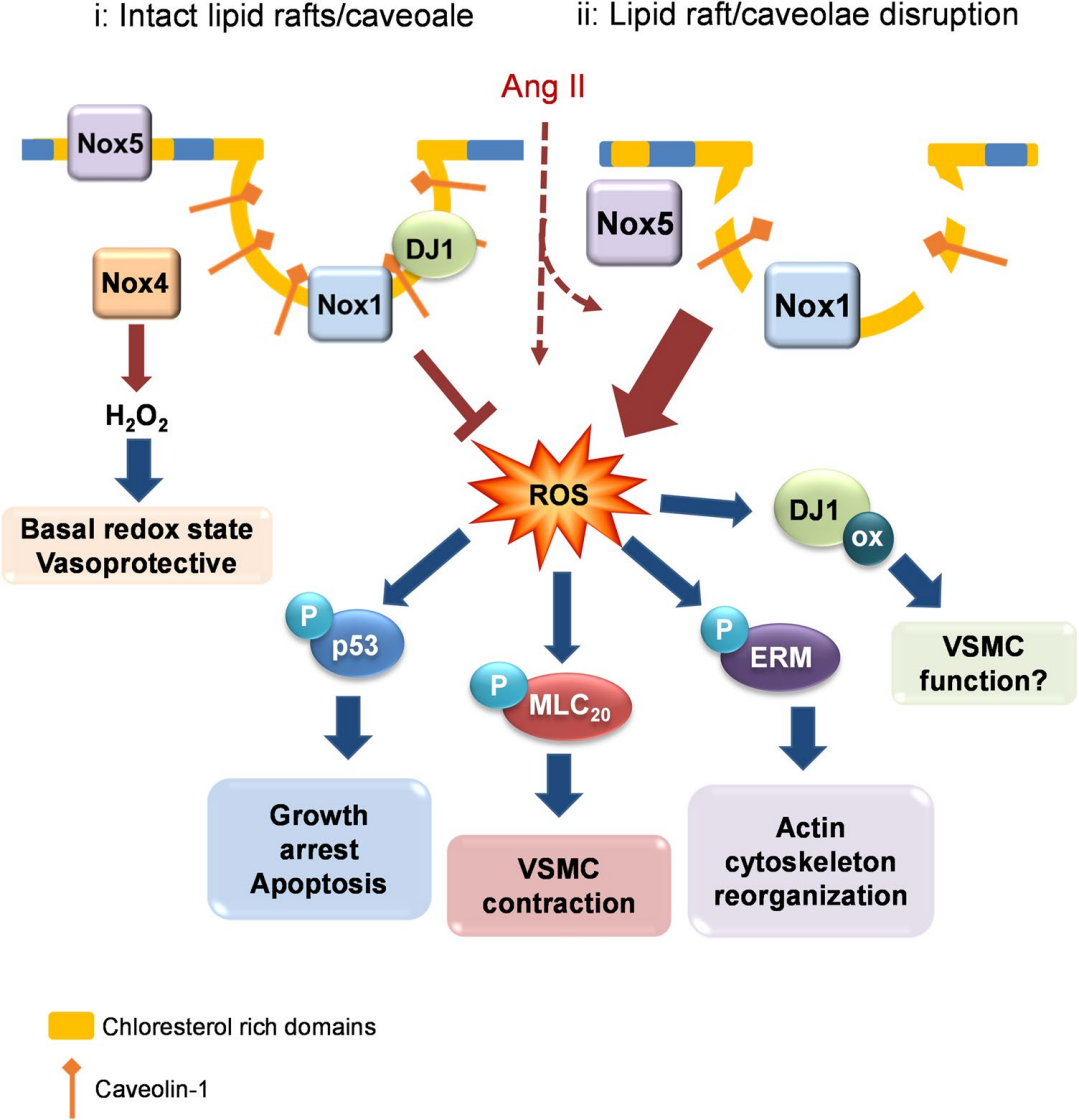
Schematic showing putative interactions between lipid rafts/caveolae, Nox isoforms, DJ-1 and vascular signaling in conditions where lipid rafts/caveolae are intact and when they are discrupted. (i) When cholesterol-rich microdomains are intact, likely in physiological conditions, Nox1 and Nox5 are regulated and ROS production is controlled. Nox4 which is constitutively active, localizes in the cytoplasm where it generates mainly H_2_O_2_, which may be vasoprotective. (ii) During lipid-raft disruption, which may occur in pathological conditions or when the Ang II system is upregulated, Nox1 and Nox5 are activated leading to excessive ROS production and oxidative stress. This promotes increased redox-dependent signaling through multiple downstream pathways that influence VSMC function. *P* phosphorylation, *Ox* oxidation, *MLC20* myosin light chain, *ERM* Ezrin–Radixin–Moesin.

Vascular signalling is mediated in large part through Nox-derived ROS and activation of redox-sensitive pathways that regulate VSMC function. Sub-cellular mechanisms underlying these processes have not been fully elucidated but compartmentalization in cholesterol-rich microdomains may be important. This is especially relevant for Ang II since the AT_1_R and associated signalling molecules localize in lipid rafts/caveolae. In the present study, using a multidisciplinary approach, we identify these microdomains as an important structural element in Nox-ROS regulation and redox-dependent signalling in human VSMCs and show that disruption of these microdomains promotes oxidative stress and aberrant vascular signalling. Our findings demonstrate that these processes are highly regulated and Nox isoform-specific since Nox1 and Nox5, but not Nox4, localize in lipid rafts/caveolae, which when disrupted lead to increased Nox-derived ROS production and hyperactivation of signalling pathways important in VSMC function including, MLC20, Ezrin–Radixin–Moesin and p53 involved in contraction, cytoskeletal organization and apoptosis/cell cycle control respectively. In addition, we identified DJ1 as a redox-sensitive downstream target regulated by Ang II in a Nox- and lipid raft/caveolae-dependent manner. To recapitulate our findings in human VSMCs in an intact system, we studied mice deficient in Cav-1 and caveolae and demonstrated that Nox-derived ROS and vascular signalling are exaggerated. Together our findings identify an important role for cholesterol-rich microdomains that act as regulatory elements for Nox1 and Nox5 and redox-sensitive signalling platforms in VSMCs (Fig. 12). Loss of integrity of these microdomains promotes oxidative stress and altered signaling, important in vascular dysfunction associated with pathological processes.

Noxs are widely recognised as key sources of ROS in vascular cells that play a crucial role in the progression of cardiovascular diseases^36, 37^. Despite advances in redox research, molecular mechanisms regulating Nox activity and their compartmentalization in the vascular system, especially in human VSMCs, is still poorly defined. This is particular relevant for Nox5, where progress has been slow due to the lack of research tools and experimental models, as *NOX5* gene is not expressed in rodents, the major experimental model used to study cardiovascular Nox biology. Although it has been proposed that Nox and ROS compartmentalize in different cellular organelles, including mitochondria and endoplasmic reticulum^38, 39^, where distinct redox signaling is tightly controlled, the role of cholesterol-rich microdomains remains unclear, particularly in the human context. Previous studies using qualitative approaches showed Nox1 in caveolae in human VSMCs^3^. In lung fibroblasts and heterologous expression systems in COS-7 expressing Cav-1/Nox5, Cav-1/Nox2, Cav-1/Nox4^11^, Nox2 and Nox5, but not Nox4, were found to associate with lipid-rafts through direct interactions with Cav-1. Using multiple approaches including pharmacological inhibitors and siRNA to downregulate Nox isoforms, we show both qualitatively and quantitatively that in human VSMCs, Nox1 and Nox5, but not Nox4 are present in cholesterol-rich fractions and that redox signalng is differentially regulated by distinct Nox isoforms. To further confirm these findings we performed co-immunoprecipitation assays to examine whether Nox1 or Nox5 directly interact with Cav-1 and showed that Nox1 co-immunoprecipitates with Cav-1. Nox5, although present in cholesterol-rich microdomains, did not interact with Cav-1, suggesting that it may associate with non-caveolae lipid fractions.

Reasons why cholesterol-rich microdomains are enriched in Nox1 and Nox5, but not Nox4, are unclear but might relate, at least in part, to different functions of these Nox isoforms. In particular, activation of Nox1 and Nox5 generate primarily O_2_^−^ whereas activation of Nox4 produces mainly H_2_O_2_^40–43^. This was corroborated in our studies in Nox5 downregulated cells, where Ang II stimulation increased production of H_2_O_2_ but not O_2_^−^. It may be possible then that O_2_^–^ generating Noxs localise in microdomains where they directly influence redox-sensitive signaling molecules, while Nox4-generating H_2_O_2_ localizes mainly in cholesterol-poor fractions, namely the cytoplasm. This differential compartmentalization may contribute to distinct O_2_^−^ versus H_2_O_2_-mediated signaling in VSMCs. This is relevant because these reactive species influence different signalling pathways with variable functional responses. For example, O_2_^−^ is associated with vasoconstriction, whereas H_2_O_2_ contributes to basal redox status and promotes vasodilation^14, 15, 17, 18^. Moreover processes of Nox regulation may be important because Nox1 and Nox5, localised in cholesterol-rich domains, are activated by vasoactive agents, whereas Nox4, which does not associate with lipid rafts/caveolae, is constitutively active.

Ang II signaling involves Nox-derived ROS as we previously demonstrated^1, 37^. Considering the importance of lipid rafts/caveolae in Nox1/5-ROS regulation in VSMCs we questioned whether Ang II influences lipid raft/caveolae trafficking of these Noxs. Within 5 min of Ang II stimulation, Nox1 shuffled from non-lipid raft fractions to lipid-rich fractions, while Nox5 moved in the opposite direction, indicating that Ang II regulates Nox trafficking in a highly organized and isoform-specific manner. The significance of this may relate to the differential functions of Nox1 and Nox5. Nox1 is important in vascular inflammation and expression/activation of pro-inflammatory adhesion molecules^44–46^, which associate with the cell membrane and lipid rafts/caveolae, while Nox5 is involved in VSMC contraction, proliferation and cytoskeletal organization^2, 23^. To dissect out distinct roles for Nox1 and Nox5 in human VSMCs we used a multidisciplinary approach including various pharmacological inhibitors (NoxA1ds, GKT137831 and mellitin) and siRNA targeted to Nox5 and p22phox-dependent Noxs, which includes Nox1-Nox4. Our findings suggest that Noxs regulate multiple signaling pathways and that Nox5 seems to be especially important in Ang II-stimulated ERK1/2 signaling whereas Nox1 is involved in Ang II-induced activation of p53. These phenomena may contribute, at least in part, to the diverse vascular actions of Ang II. It should be highlighted that we assessed acute signaling events and that different Nox-dependent responses may occur with chronicstimulation.

Lipid-rafts/caveolae seem to play an important regulatory role in maintaining basal ROS production, because disruption of these microdomains or depletion of cholesterol, caused excessive ROS generation, processes that lead to oxidative stress, cell injury and vascular dysfunction. This is exemplified by our findings that in human VSMCs exposed to MCD and nystatin, Nox-derived ROS generation was increased, findings that were recapitulated in Cav-1-silenced human VSMCs and vessels from Cav-1^−/−^ mice. Similar responses have been observed in MCD-treated mouse macrophages, macrophages from Cav-1^−/−^ mice and in human lung fibroblasts where Cav-1 was knocked down by siRNA^11^. These results together with our findings support the notion that cholesterol-rich microdomains are negative regulators of Nox-derived O_2_^−^ generation. While we can not distinguish exactly which Nox isoform is involved in this process, it is likely to be Nox1 and/or Nox5, but not Nox4 because Nox4 does not seem to be abundant in lipid-rafts and Nox4 generates primarily H_2_O_2_.

Vascular signaling through lipid rafts/caveolae is highly selective. For example, phosphorylation of MLC20, Ezrin/Radixin/Moesin and p53, but not ERK1/2, was variably increased. ERK1/2 activation seems to be independent of cholesterol microdomains in human VSMCs. This was corroborated in VSMCs in which Cav-1 was downregulated, since phosphorylation of Ezrin/Radixin/Moesin and p53, but not ERK1/2, was increased. These in vitro results are in agreement with intact vessels isolated from Cav-1^−/−^ mice where phosphorylation levels of ERK1/2 were similar to vessels from wild-type mice. The importance of Cav-1 in ERK1/2 activation remains controversial, as previous studies showed that Cav-1 might have an inhibitory or stimulatory role depending on the cell type. In rat aortic VSMCs, cardiac fibroblasts, NIH 3T3 fibroblast and Rat-1 cells, regulation of ERK1/2 activity is Cav-1-dependent^47–50^, whereas in bovine aortic endothelial cells and in mouse embryonic fibroblasts it seems to be Cav-1-independent^7, 51^. On the other hand, signalling pathways associated with cell contraction and cytoskeletal organisation in human VSMCs seem to be controlled by microdomain integrity, because lipid-raft disruption induced phosphorylation of MLC20 in human VSMCs, a response that is Nox5-dependent as we previously demonstrated^23^. The importance of lipid rafts/caveolae in the regulation of vascular contraction has been demonstrated in rat tail artery rings and rat cremaster arterioles^52, 53^ and confirmed in aortic rings from Cav-1^−/−^ mice, which exhibited reduced contractile responses to vasoconstrictive agents including Ang II, endothelin-1 and phorbol ester^6^.

Cholesterol-rich microdomains interact with the cell cytoskeleton and are intricately involved in the organisation of the actin and its associated structural proteins^54^. Cholesterol depletion promotes F-actin polymerisation and stress fibre formation in tumour cells^55^, mesenchymal and epithelial cells while in fibroblasts MCD induces actin disassembly and reduction of stress fibres^56^. In our study we showed that cholesterol-rich microdomains influence activation of the actin cytoskeleton- associated proteins, Ezrin/Radixin/Moesin, which act as cross-linkers between plasma membranes and actin filaments. Phosphorylation of these proteins is necessary for its binding to the F-actin cytoskeleton^57, 58^. We found that lipid-raft disruption and Cav-1 downregulation in human VSMCs was associated with increased phosphorylation of Ezrin/Radixin/Moesin indicating the importance of intact microdomains for cytoskeleton organization. These findings were further confirmed in intact vessels from Cav-1^−/−^ mice.

Another signaling system regulated by lipid rafts/caveoale involves p53, a master transcriptional factor controlling genes involved in cell cycle arrest, senescence or apoptosis. Phosphorylation of p53 at Ser15 and Ser20 disrupts the interaction between p53 and its negative regulator MDM2, leading to the accumulation and activation of p53 in response to cellular stress-induced DNA damage^59, 60^. We found that Ang II increased phosphorylation of p53 (Ser 15) in human VSMCs, similar to what has been shown in neonatal rat cardiomyocytes, H9c2 cells^61^ and rat aortic cells^62, 63^. We also found that MCD increased phosphorylation of p53 (Ser 15) supporting a role for cholesterol-rich fractions in p53 regulation, similar to what was demonstrated in chicken myogenic cells^64, 65^. To further support this notion, ROS-induced activation of p53 in fibroblasts from Cav-1^−/−^ mice was altered^66, 67^ and transient overexpression of Cav-1 in mouse NIH 3T3 fibroblasts or stable transgenic Cav-1 expression in fibroblasts caused cell cycle arrest and senescence^68^.

In addition to identifying a regulatory role for microdomains in Nox-ROS-redox signalling, we found that DJ1 is a caveolar-resident protein that interacts with Cav-1 and Nox1 in human VSMCs. DJ1 is increasingly being recognized as an important redox-sensitive molecule involved in vascular regulation^66–68^. DJ1 acts as a ROS scavenger and also as a signaling protein that activates transcriptional factors, such as nuclear factor erythroid 2-related factor 2 (Nrf-2)^26, 69^. The anti-oxidant function of DJ1 depends on its oxidation state of Cys-106, which is oxidised by ROS from sulfenic acid (Cys-SOH) to sulfinic acid (Cys-SO_2_H) to sulfonic acid (Cys-SO_3_H). Having observed that DJ1 is abundantly expressed in human arteries and VSMCs, we questioned whether DJ1 may be another redox-sensitive target regulated by Ang II through Nox-lipid raft/caveolae mechanisms. We found that Ang II rapidly increased irreversible oxidation of DJ1 (Cys 106-SO_2_H; Cys 106-SO_3_H), but only in the non-lipid raft fraction. To our knowledge these are the first studies showing that Ang II regulates DJ1 in VSMCs through processes involving rafts and oxidation. The functional significance of this awaits clarification.

Similar to many other studies^70–74^, we used a pharmacological approach to disrupt lipid rafts/caveolae in human VSMCs. However this approach does have limitations because MCD has been shown to disrupt cholesterol-rich domains beyond the plasma membrane^70, 71^. In particular MCD influences membranes of subcellular organelles, including mitochondria, and accordingly we can not exclude the potential role of other sources, such as mitochondria, for ROS in our experimental paradigm. To mitigate some of these limitatons, we used a second disrupter, nystatin to interrogate lipid rafts. Moreover we studied VSMCs in which Cav-1 was downregulated using siRNA approaches.

In conclusion, we demonstrate that Nox1 and Nox5 localize and traffic through cholesterol-rich microdomains, which act as negative regulators for Nox-induced ROS generation and redox signaling in human VSMCs. Loss of integrity of cholesterol-rich microdomains promotes oxidative stress and alters signaling important in vascular dysfunction associated with cardiovascular disease. Our findings suggest that lipid rafts/caveolae are discrete sub-cellular compartments involved in Nox1 and Nox5-derived ROS generation and signaling in human VMSCs. This regulatory system is Nox isoform-specific because Nox4-derived H_2_O_2_ seems to be independent of lipid rafts/caveolae.

## Methods

Detailed methodology is provided in the Online Data Supplement.

### Human vascular tissue and primary human vascular smooth muscle cell culture

All studies related to accessing small arteries and vascular smooth muscle cells (VSMCs) from humans were approved by the West of Scotland Research Ethics Service (WS/12/0294). Written informed consent was received from all study participants in accordance with the Declaration of Helsinki (1997). Human small arteries were obtained from patients undergoing elective craniofacial surgeries (n = 12) at the Queen Elizabeth University Hospital, Glasgow. A small piece of vascular tissue was fixed in 4% paraformaldehyde (PFA) overnight and used for immunofluorescence. The remaining tissue was used to isolate VMSCs by enzymatic digestion, as previously described^39, 75^. Experiments were performed on low-passage cells (passage 4–6).

VSMCs were serum-deprived overnight and stimulated with 100 nmol/L Ang II for 5 and 15 min. In some studies, cells were pre-treated with lipid-raft disruptors methyl-β-cyclodextrin (MCD, 10 mmol/L, Sigma Aldrich, 332615) for 45 min or Nystatin (Nys, 50 μg/mL, Sigma Aldrich, N3503) for 30 min and then stimulated with Ang II. To investigate the role of Noxs in VSMC signalling, cells were pre-treated (30 min) with NoxA1ds (Nox1 inhibitor, 10 μmol/L, Merck 532759), GKT137831 (Nox1/NOX4 inhibitor, 10 μmol/L, Cambridge Bioscience CAY17764) or melittin (Nox5 inhibitor, 100 nmol/L; Sigma-Aldrich M2272). Concentrations used were based on previously published data^33–35, 39^.

### Immunofluorescence and immunohistochemistry staining of human small arteries

Immunofluorescence of Nox5, Nox4, total DJ1 and Cav-1 and immunohistochemistry of irreversibly oxidized DJ1 (Cys-SO_2_H and Cys-SO_3_H) was determined in human small arteries.

### Isolation of cholesterol-rich microdomain by protein fractionation using detergent-free sucrose gradient centrifugation

Lipid-rafts/caveolae in VSMCs stimulated with/without Ang II for 5, 15 or 30 min were isolated by protein fractionation using the detergent-free sucrose gradient centrifugation method as we previously described^73, 74^. Fractionated proteins from VSMCs were separated by immunoblotting. In some experiments, isolated lipid-raft fractions (fractions 3–4) and non-lipid-raft fractions (7–12) were pooled together.

### Cholesterol depletion/sequestration and cholesterol reloading

To disrupt caveolae in VSMCs, free cholesterol was depleted or sequestrated from the plasma membrane by using two different agents, MCD or Nys, respectively as previously described^74^. Cholesterol was depleted by treating VSMCs with mM MCD for 45 min at 37 °C. Cholesterol was sequestrated by treating VSMCs with 50 μg/mL Nys for 30 min. In some experiments, after MCD treatment, cholesterol was reloaded with cholesterol:MCD (1–10 mmol/L) complex.

### Immunoblotting

Total or fractionated proteins from VSMCs were separated by electrophoresis on a polyacrylamide gel, transferred onto a nitrocellulose membrane and probed with primary antibodies. Horseradish peroxidase-conjugated secondary antibodies were visualised by Azure c300 Western Blot Chemiluminescent Blot Imaging System and fluorescent-conjugated secondary antibodies were visualised by infrared laser scanner. Bands were quantified densitometrically with either ImageJ (https://imagej.nih.gov/ij/) or Image Studio Lite software from LI-COR.

### Co-immunoprecipitation and immunoblotting

Immunoprecipitation was performed on 200–500 μg protein lysates from VSMCs using specific primary antibodies as previously described^76^. Immunoprecipitates were resolved in SDS–polyacrylamide gels. The following antibodies were used for immunoprecipitation (IP): anti-mouse Cav-1 (Bd Biosciences), anti-rabbit Total DJ1 (Abcam), anti-rabbit Nox5 (a generous gift from Professor William Nauseef) and anti-goat Nox1 (Sigma-Aldrich). For Cav-1 and DJ1 IP, the immunoblottings were performed with anti- rabbit Cav-1 (Cell Signaling, 3267), anti-rabbit Nox1 (Sigma-Aldrich, SAB4200097), anti-rabbit Nox5 and anti-rabbit DJ1. For Nox5 IP, the immunoblottings were performed with anti-mouse Cav-1, anti-rabbit DJ1 and anti-rabbit Nox5. For Nox 1 IP, the immunoblottings were performed with anti-mouse Cav-1, anti-rabbit DJ1 and anti-rabbit Nox1. WB Optima A or B or C or F detection systems (Santa Cruz Biotechnology, sc-4503 or sc-45038 or sc-45039 or sc-45040 respectively) were used to prevent interference of IgG chains in the immunoblot assays.

### Immunocytochemistry

Human VSMCs were cultured on sterile glass coverslips, fixed in ice-cold 100% methanol for 5 min, blocked with 3% Bovine Serum Albumin (BSA) and incubated with primary antibodies overnight at 4 °C. Proteins were detected with Alexa Fluor secondary antibodies and slides were mounted in ProLong Gold anti-fade mounting media containing DAPI (Life technologies) overnight at RT. Fluorescence imaging was recorded in an Axiovert 200M microscope with a laser scanning module LSM 510 (Carl Zeiss AG, Heidelberg, Germany).

### Hydrogen peroxide measurement—Amplex Red Assay

Hydrogen peroxide production in VSMC lysate was measured using the horseradish persoxidase-linked Amplex Red™ Hydrogen Peroxide/Peroxidase Assay Kit according to manufacturer’s instructions (Life Technologies, Paisley, UK).

### Lucigenin-enhanced chemiluminescence

The lucigenin-enhanced chemiluminescence assay was used to assess NAD(P)H-dependent superoxide anion (O_2_^−^) production in VSMC homogenates as previously described^39^.

### Cav-1, Nox5 and p22phox downregulation with siRNA

Human VSMCs were transfected with 10 nmol/L of Cav-1 siRNA (Silencer® Select siRNA, Thermo Fischer Scientific), 20 nmol/L of p22phox siRNA (Stealth RNAi™ siRNA, Thermo Fischer Scientific) or 50 nmol/L of Nox5 siRNA (Stealth RNAi™ siRNA, Thermo Fischer Scientific) complexed with Lipofectamine™ RNAiMAX (Thermo Fischer Scientific). A sequence not homologous to any gene in the vertebrate transcriptome was used as control siRNA (Stealth RNAi™ siRNA Negative Control Lo GC, Thermo Fischer Scientific).

### Statistical analysis

Statistical analysis was performed using GraphPad Prism 8.0 (GraphPad Software Inc, San Diego, CA, USA). All data are expressed as mean ± SEM. Statistical comparisons were made with one-way ANOVA followed by Newman–Keuls test or 2-tailed Student’s *t* test as appropriate. *P* < 0.05 was considered statistically significant.

## Supplementary information

Supplementary Information.

## Data Availability

The datasets generated during and/or analysed during the current study are available from the corresponding author on reasonable request.
