# Supplementary material for: Importance of cholesterol-rich microdomains in the regulation of Nox isoforms and redox signaling in human vascular smooth muscle cells
Source: Sci Rep. 2020 Oct 20;10:17818. doi: 10.1038/s41598-020-73751-4 (PMC7575553; doi:10.1038/s41598-020-73751-4)

MS # SREP-19-06437

SUPPLEMENTARY TEXT

Importance of cholesterol-rich microdomains in the regulation of Nox isoforms and redox signaling in human vascular smooth muscle cells

*Aikaterini Anagnostopoulou PhD, *Livia De Lucca Camargo PhD, Daniel Rodrigues BSc, Augusto C Montezano PhD, Rhian M Touyz MD, PhD

Institute of Cardiovascular and Medical Sciences, British Heart Foundation Glasgow Cardiovascular Research Centre, University of Glasgow, 126 University Place, Glasgow, G12 8TA, UK

*First co-authors

Correspondence should be addressed to:
Rhian M Touyz MD, PhD
Institute of Cardiovascular and Medical Sciences,
BHF Glasgow Cardiovascular Research Centre,
University of Glasgow,
126 University Place,
Glasgow,
G12 8TA
rhian.touyz@glasgow.ac.uk
Tel: 0044 141 330 7775/7774

Methods

Human vascular tissue and primary human vascular smooth muscle cell culture

All studies related to accessing small arteries and vascular smooth muscle cells (VSMCs) from humans were approved by the West of Scotland Research Ethics Service (WS/12/0294). Written informed consent was received from all study participants in accordance with the Declaration of Helsinki (1997). Human small arteries (150-300 μm) were obtained from excess surgical tissue (subcutaneous tissue from the neck or face) from patients undergoing elective craniofacial surgeries (n=12) at the Queen Elizabeth University Hospital, Glasgow. A small piece of vascular tissue was fixed in 4% paraformaldehyde (PFA) overnight and used for immunofluorescence studies. The remaining tissue was used for isolation of primary VSMCs as we previously described[1]. All reagents were purchased from Sigma-Aldrich unless otherwise specified. Experiments were performed on low-passage cells (passage 4-6).

VSMCs were grown in culture medium containing DMEM supplemented with Smooth Muscle Growth Supplement (25 ml, SMGS, Gibco, S-007-25), Penicillin/Streptomicin (50 μg/ml, Gibco, 15140-122). For all experiments, 80-90% confluent cells were rendered quiescent by serum deprivation (0.5% FBS in DMEM) for 18 hrs. The following day, VSMCs were stimulated with 100 nmol/L Ang II for 5 and 15 mins. In some studies, cells were pre-treated with lipid-raft disruptors methyl-β-cyclodextrin (MCD, 10 mmol/L, Sigma Aldrich, 332615) for 45 mins, Nystatin (Nys, 50 μg/ml, Sigma Aldrich, N3503) for 30 mins and then stimulated with Ang II. To investigate the role of NOXs in VSMC signalling cells were pre-treated (30 mins) with NOXA1ds (NOX1 inhibitor, 10 μmol/L, Merck 532759), GKT137831 (NOX1/NOX4 inhibitor, 10 μmol/L,Cambridge Bioscience CAY17764) or melittin (NOX5 inhibitor, 100 nmol/L; Sigma-Aldrich M2272).

Immunofluorescence and immunohistochemistry staining of human small arteries

Immunolocalization of Nox isoforms, DJ1 and Cav1 was determined in human small arteries. Briefly, 4 μm paraffin-sections of arteries were deparaffinised in xylene (3 x 7 min) and rehydrated in descending series of ethanol solutions (100%, 100%, 95%, 95%, 70% and 50%) for 5 mins each, washed in dH_2O for 5 min, followed by a final rinse in Tris-buffered saline (TBS) solution for 5 mins. Antigen retrieval was performed by boiling the slides in 1 mM EDTA (pH 8.0) for 15 mins. After cooling the slides at room temperature (RT) for 30 mins, the slides were washed three times with TBS for 5 mins each. Sections were blocked in 10% goat serum, 1% BSA in TBS for 1 hr at RT in a humidified chamber. After blocking, sections were incubated with primary antibodies overnight at 4^0C in a humidified chamber. Primary antibodies used were as follows: rabbit polyclonal anti-Nox5 customised antibody[2] (1:75, a generous gift from Professor David J. Lambeth, University of Emory, Atlanta, U.S.A.), mouse monoclonal anti-Cav1 (1:100, Bd Biosciences, 610407), rabbit monoclonal anti-DJ1 (1:200, Abcam, ab76008), human monoclonal anti-oxidised DJ1 (Cys 106-SO_3H; 1:200, Bio-rad, AbD03055), rabbit polyclonal anti-Nox4 (1:100, Abcam, ab133303). For negative controls, rabbit (Cell Signalling, 3900) or mouse (Cell Signalling, 5415), IgG matched isotype controls were used. The following day, the sections were washed three times with TBS for 10 min each. For immunofluorescence staining of Nox5, Nox4, total DJ1 and Cav1, sections were incubated with Alexa-fluor-488 goat anti-rabbit or Alexa-fluor-555 goat anti-mouse conjugated secondary antibody (1:300; Life technologies, Molecular Probes) for 45 min at RT in the dark, followed by three washes in TBS for 10 mins. Primary and secondary antibodies were diluted in 5% goat serum, 0.1% BSA in 1xTBS. Slides were mounted in ProLong Gold anti-fade mounting media containing DAPI (Life technologies, Molecular Probes P-36931) overnight at RT. Fluorescence imaging was recorded in an Axiovert 200M microscope with a laser scanning module LSM 510 (Carl Zeiss AG, Heidelberg, Germany) using a 40x objective. DAPI was excited at 405 nm, Alexa-fluor 488 at 488 nm and Alexa-fluor 555 at 543 nm. Images were recorded using the 'Physiology Evaluation' software package (Zeiss) and image processing was performed using Image J.

To detect the irreversibly oxidised form of DJ1 (DJ1 Cys 106-SO_2H; DJ1 Cys 106-SO_3H) sections were incubated with anti-human oxidised DJ1 (Cys106) tagged with a myc-tag at the C-terminus of the antibody (1:200, Bio-Rad AbD Serotec, clone AbD03055, HCA024) overnight at 4^0C in a humidified chamber. This antibody was synthesised using a synthetic peptide containing SO_3H at Cys106 and recognises both irreversibly oxidised forms of DJ1 (Cys106 SO_2H and SO_3H) [3]. The following day, the sections were washed three times with TBS for 10 min each and incubated with mouse monoclonal anti-human Myc tag antibody conjugated with horseradish peroxidase (1:500, 9E101, Abcam, ab62928) for 1 h at RT. IHC staining was detected by incubating the sections with DAB peroxidase solution (Vector Labs, SK-4100) in the presence of H_2O_2 for 5 mins. Nuclei were counterstained with Harris haematoxylin for 1 min; followed by dehydration of sections. Slides were mounted using DPX-mounting medium and imaging was recorded under an Axio Observer Z.1 transmitted-light inverted light microscope with a digital colour camera.

Isolation of cholesterol-rich microdomain by protein fractionation using detergent-free sucrose gradient centrifugation

Lipid-rafts/caveolae in VSMCs stimulated with/without Ang II for 5, 15 or 30 mins were isolated by protein fractionation using the detergent-free sucrose gradient centrifugation method as we previously described [4]. Briefly, Ang II-or vehicle stimulated VSMCs were lysed in 500 mmol/L Na_2CO_3 (pH 11). 2 mg of protein homogenate was adjusted to 45% sucrose in 2-(*N*-morpholino) ethanesulfonic acid (MES)-buffered saline (MBS) (25 mmol/L MES and 150 mmol/L NaCl, pH 6.5) and overlayered with 6 mL of 35% sucrose and 2 mL of 5% sucrose. After centrifugation at 37 000 g for 18 h at 4^0C (SW 41, Beckman Instruments), twelve 1-mL fractions were collected. Proteins were precipitated with 10% trichloroacetic acid and re-suspended in 0.1 mol/L NaOH and Laemmli buffer. Caveolin-1 (Cav1) was used as a marker of lipid rafts. Fractionated proteins from VSMCs were separated by immunoblotting. In some experiments, isolated lipid-raft fractions (fractions 2-4) and non-lipid-raft fractions (7-12) were pooled together.

Cholesterol depletion/sequestration and cholesterol reloading

To disrupt caveolae in VSMCs, free cholesterol was depleted or sequestrated from the plasma membrane by using two different agents, MCD or Nys, respectively, as previously described [5]. Briefly, cholesterol was depleted by treating VSMCs with 10 mM MCD in DMEM for 45 mins at 37^0C. Cholesterol was sequestrated by treating VSMCs with 50 μg/ml nystatin in DMEM for 30 mins. Following MCD or Nys treatment, VSMCs were washed with DMEM to remove MCD or Nys and immediately used for experiments. In some experiments, after MCD treatment, cholesterol (water-soluble, Sigma-Aldrich C4951) was reloaded with cholesterol:MCD (1:10 mM) complex in DMEM for 45 mins at 37^0C as previously described[5].

Immunoblotting

Total or fractionated proteins from VSMCs were separated by electrophoresis. Briefly, VSMCs were washed twice with ice-cold PBS, lysed in protein lysis buffer (50 mmol/L sodium pyrophosphate, 50 mmol/L NaF, 5 mmol/L NaCl, 5 mmol/L EDTA, 5 mmol/L EGTA, 10 mmol/L HEPES , 2 mmol/L Na_3VO_4, 50 mmol/L PMSF, 0.5%Triton-X 100 and 1 mg/mL leupeptin/aprotinin/pepstatin) and sonicated for 5 seconds. Proteins extracted from each lysate (30 μg) were separated by electrophoresis on a 7.5, 10 or 12 % SDS polyacrylamide gel and transferred to a nitrocellulose membrane. Non-specific binding sites were blocked with 5% milk in TBS solution with 0.1% Tween-20 (TBST) for 1 hour at RT. Membranes were incubated with primary antibodies (1:1000 dilution) overnight at 4^0C. The following day, membranes were washed three times for 10 mins in TBST and incubated with horseradish peroxidase-conjugated secondary antibodies (Jackson ImmunoResearch Laboratories) or IRDye Infrared Dye (680 LT or 800 CW) fluorescent-conjugated secondary antibodies (LI-COR Biotechnology). Signals from membranes incubated with peroxidase-conjugated secondary conjugated antibodies were revealed by enhanced chemiluminescence (WestPico, Pierce) and visualized by Azure c300 Western Blot Chemiluminescent Blot Imaging System. Bands from

membranes incubated with IRDye Infrared fluorescent- conjugated secondary antibodies were visualized using the LI-COR Odyssey CLx Imaging System. Bands were quantified densitometrically with either ImageJ (http://imagej.nih.gov/ij/) or Image Studio Lite software (LI-COR Biotechnology). Results were normalized to β-actin or α-tubulin. Primary antibodies used were as follows: mouse anti-β -actin (Abcam, ab8229); rabbit anti-α-tubulin (Abcam, ab18251); rabbit anti-phospho-Ezrin-Radixin-Moesin (Cell Signaling, 3141S); rabbit anti-phospho-p44/42 MAPK (ERK1/2; Cell Signaling, 9101); mouse anti-p44/42 MAPK (ERK1/2; Santa Cruz, 514302); rabbit anti-phospho-MLC20 (Cell Signaling, 3674S); rabbit anti-phospho p53 (Cell Signalling, 9284); mouse anti-Cav1 (Bd Biosciences, 610407); rabbit anti-DJ1 (Abcam, ab76008); rabbit anti-oxidised DJ1 (Cys 106-SO_3H; Abcam, ab169520); goat anti-Nox1 (Sigma-Aldrich, SAB 2501686); rabbit anti-Nox5[6] (provided by Professor William Nauseef, University of Iowa, Iowa, U.S.A.); rabbit anti-Nox4 (Abcam, ab133303); rabbit anti-p22phox (Abcam, ab75941).

Co-immunoprecipitation and immunoblotting

After MCD or Nys treatment, VSMCs were washed twice with ice-cold PBS, scraped using HEPES buffer and homogenized by passing the cells through a 22G needle syringe 20 times. VSMCs homogenates were adjusted to equal protein concentration (1 µg/µl), and 200-500 µg of protein was used for the assay. Cell lysates were pre-cleared with 40 µl Protein G Plus/A agarose IP beads (Merck Millipore, IP02/IP04) for one hour at 4^0C. 5 µg of immunoprecipitating antibody was added to 40 µl of Protein G plus/A Agarose IP suspension and 500 µl of HEPES buffer and incubated on a rotor overnight at 4^0C. The following day, the antibody/beads complex was washed three times in HEPES buffer for 30 sec at maximum speed at 4^0C. After the final wash, cell lysates were transferred to the beads and incubated on a rotor overnight at 4^0C. The following day, the beads with the immunocomplexes were washed three times with HEPES buffer for 30 sec at maximum speed at 4^0C. The beads were resuspended in 50 µl of 2x sample buffer and boiled for 5 mins to dissociate the immunocomplexes from the beads. Immunoprecipitates were resolved in SDS-polyacrylamide gels. The following antibodies were used for immunoprecipitation (IP): mouse anti-Cav1 (Bd Biosciences, 610407), rabbit anti-DJ1 (Abcam, ab76008), rabbit anti-Nox5[6] (a generous gift from Professor William Nauseef) and goat anti-Nox1 (Sigma-Aldrich, SAB 2501686). WB Optima A, B, C or F detection systems (Santa Cruz Biotechnology; sc-4503, sc-45038, sc-45039 or sc-45040 respectively) were used to prevent interference of IgG chains in the immunoblot assays.

Immunocytochemistry

Human VSMCs were plated in sterile glass coverslips and once reached 70-80% confluence, they were serum deprived with 0.5% FBS in DMEM overnight. The next day, VSMCs were fixed in ice-cold 100% methanol for 5 mins, followed by three washes of PBS for 5 mins. Fixed VSMCs were blocked with 3% Bovine Serum Albumin (BSA) for 1 hour at RT and incubated with the primary antibodies against goat anti-Nox1 (Sigma-aldrich) and rabbit anti-DJ1 (Abcam) overnight at 4^0C. Following incubation, VSMCs were washed three times with PBS for 5 mins each and incubated with Alexa-fluor-488 donkey anti-rabbit or Alexa-fluor-555 donkey anti-goat conjugated secondary antibody (1:300; Life technologies, Molecular Probes) for 45 min at RT in the dark. After inclubation, cells were washed 3x in TBS for 10 mins. Primary and secondary antibodies were diluted in 3% BSA in PBS. Slides were mounted in ProLong Gold anti-fade mounting media containing DAPI (Life technologies) overnight at RT. Fluorescence imaging was recorded in an Axiovert 200M microscope with a laser scanning module LSM 510 (Carl Zeiss AG, Heidelberg, Germany) using a 40 x objective. DAPI was excited at 405 nm, Alexa-fluor 488 at 488 nm and Alexa-fluor 555 at 543 nm. Images were recorded using the 'Physiology Evaluation' software package (Zeiss) and image processing was performed using Image J.

Hydrogen Peroxide measurement - Amplex Red Assay

Hydrogen peroxide production in VSMC lysate was measured using the horseradish peroxidase linked Amplex Red™ fluorometric assay (Life Technologies, Paisley, UK) according to

manufacturer's instruction. Briefly, VSMCs were lysed in the same protein lysis buffer used for immunoblotting. Amplex Red (5 µmol/L) and horseradish peroxidase (0.1 U/mL) were added to 50 µl of VSMC lysate. Fluorescence readings were made in a 96-well plate at Ex/Em of 530/590 nm. H_2O_2 levels were calculated based on standard H_2O_2 curve and normalized to protein concentration for each sample, measured by BCA assay (Thermo Fisher, 23225). Results are expressed as a fold change in arbitrary units/µg protein.

Lucigenin-enhanced chemiluminescence

Lucigenin-enhanced chemiluminescence assay was used to assess NADPH-dependent superoxide anion (O_2^-) generation in VSMC. Briefly, stimulated cells were washed twice with ice-cold PBS and harvested in ROS phosphate buffer (50 mmol/L KH_2PO_4, 10 mmol/L EGTA, 1 µg/mL aprotinin, 1 µg/mL leupeptin, 1 µg/mL pepstatin, and 1 mmol/L PMSF). 50 µl of the sample was added to a suspension containing 175 µl of assay buffer (50 mmol/L KH_2PO_4, 10 mmol/L EGTA, and 1.5 mmol/L Sucrose) and lucigenin (5 µmol/L, Sigma-Aldrich, M8010). Luminescence was measured for 29 cycles of 1.8 seconds each by a luminometer (Lumistar Galaxy; BMG Labtechnologies). Basal readings were recorded prior to the addition of NADPH as the substrate and were subtracted from the NADPH-dependent luminescence signal. ROS production was expressed as relative light units (RLU)/µg protein, as measured by the BCA assay (Thermo Fisher, 23225).

Cav-1, Nox5 and p22phox downregulation with siRNA

Human VSMCs were plated and cultured for 24h in growth medium (DMEM supplemented with SMGS and 5% antibiotics). VSMCs were incubated with 10 nmol/L of Cav-1 siRNA (Silencer® Select siRNA, Thermo Fischer Scientific), 20nmol/L of p22phox siRNA (Stealth RNAi™ siRNA, Thermo Fischer Scientific) or 50nmol/L of Nox5 siRNA (Stealth RNAi™ siRNA, Thermo Fischer Scientific) complexed with Lipofectamine™ RNAiMAX (Thermo Fischer Scientific) as transfection reagent in DMEM without serum and antibiotics for 6 h. A sequence not homologous to any gene in the vertebrate transcriptome was used as control siRNA (Stealth RNAi™ siRNA Negative Control Lo GC, Thermo Fischer Scientific).

Statistical Analysis

Statistical analysis was performed using GraphPad Prism 8.0 (GraphPad Software Inc, San Diego, CA). All data are expressed as mean ± SEM. Statistical comparisons were made with one-way ANOVA followed by Newman–Keuls test or 2-tailed Student's *t* test as appropriate. $P<0.05$ was considered statistically significant.

References

1 Montezano, A. C., Lopes, R. A., Neves, K. B., Rios, F. & Touyz, R. M. Isolation and Culture of Vascular Smooth Muscle Cells from Small and Large Vessels. *Methods Mol Biol* **1527**, 349-354, doi:10.1007/978-1-4939-6625-7_27 (2017).

2 Brar, S. S. *et al.* NOX5 NAD(P)H oxidase regulates growth and apoptosis in DU 145 prostate cancer cells. *Am J Physiol Cell Physiol* **285**, C353-369, doi:10.1152/ajpcell.00525.2002 (2003).

3 Ooe, H., Iguchi-Ariga, S. M. & Ariga, H. Establishment of specific antibodies that recognize C106-oxidized DJ-1. *Neurosci Lett* **404**, 166-169, doi:10.1016/j.neulet.2006.05.031 (2006).

4 Callera, G. E., Bruder-Nascimento, T. & Touyz, R. M. Assessment of Caveolae/Lipid Rafts in Isolated Cells. *Methods Mol Biol* **1527**, 251-269, doi:10.1007/978-1-4939-6625-7_20 (2017).

5 Callera, G. E. *et al.* Vascular proinflammatory responses by aldosterone are mediated via c-Src trafficking to cholesterol-rich microdomains: role of PDGFR. *Cardiovasc Res* **91**, 720-731, doi:10.1093/cvr/cvr131 (2011).

6 El Jamali, A. *et al.* Novel redox-dependent regulation of NOX5 by the tyrosine kinase c-Abl. *Free Radic Biol Med* **44**, 868-881, doi:10.1016/j.freeradbiomed.2007.11.020 (2008).

Supplementary figure legends

Supplementary figure 1. Nox5 and p22phox silencing in human VSMC. A) Nox5 expression detected by western blot in cells transfected with Nox5 or control (Ctl) siRNA. B) Expression of p22phox in cells transfected with p22phox or Ctl siRNA. α-tubulin was used as loading control. Data are means ± SEM from 7-8 experiments. Control was taken as 100% and data are presented as the percentage changes relative to control conditions. *P<0.05 vs Ctl siRNA.

Supplementary figure 2. DJ1 is expressed in human small arteries. Paraffin-embedded sections of human small arteries were immunostained with DJ1. (A) Green immunofluorescence indicates DJ1 immunostaining and blue indicates nuclear staining using DAPI. Images were recorded with an Axio Observer Z.1 fluorescence inverted microscope (Carl Zeiss AG, Heidelberg, Germany) using a 40x objective. (B) Oxidized DJ1 staining was detected with DAB peroxidase and nuclei were counterstained with Harris haematoxylin. Images were recorded using an Axio Observer Z.1 transmitted light inverted light microscope (Carl Zeiss AG, Heidelberg, Germany) with a digital colour camera using a 5x (a, b) and 40x (c) objective. Image processing was obtained by Image J. Asterisk: Vascular media comprising VSMCs. Arrow heads: Endothelium. IgG indicates Immunoglobulin G; Neg: Negative; Oxi: Oxidised.

Supplementary figure 3. Protein-protein interactions of Cav-1 with Nox1 and Nox5 and DJ1 in human VSMCs in the absence or presence of lipid raft disruptors. Human VSMCs were treated with or without lipid raft disruptors: 10 mmol/L methyl-b-cyclodextrin (MCD) for 45 mins or 50 mg/ml nystatin (Nystatin) for 30 mins. Protein lysates of human VSMCs were subjected to immunoprecipitation (IP) and immunoblotting (IB) with the following antibodies: (A) IP with anti-Nox5 and IB with anti-Cav1 and anti-DJ1. Cav1 interacts with Nox1 and DJ1 which is disrupted by lipid raft disruptors in human VSMCs by Co-IP. (B) IP with anti-Cav1 and IB with anti-Nox5, anti-Nox1, anti-Cav1 and anti-DJ1. (C) IP with anti-DJ1 and IB with anti-Nox1, anti-Cav1 and anti-DJ1. (D) Immunofluorescence of DJ1 and Nox1 in human VSMCs. Green immunofluorescence indicates DJ1 immunostaining, red indicates Nox-1 staining and blue indicates nuclear staining using DAPI. After probing with the respective antibodies, slides were mounted, images were recorded in an Axiovert 200M microscope with a laser scanning module LSM 510 (Carl Zeiss AG, Heidelberg, Germany) using a 40x objective. DJ-1 colocalises with Nox1 (white arrow) by ICC**.** Representative immunoblots of Nox5, Nox1, DJ1 and Cav1 are from at least 3 separate experiments. Cav1 indicates Caveolin 1; ICC: Immunocytochemistry; IgG: Immunoglobulin G; Inp: Input; IP: Immunoprecipitation; LR: Lipid Rafts; NLR: Non-Lipid Rafts; M: Marker; MCD: Methyl-b-cyclodextrin; Nox1, 4, 5: NADPH oxidase 1, 4, 5; Nys: Nystatin; WB: Western Blotting; Veh: Vehicle.

Supplementary figure 4. Cav-1 silencing in human VSMC. A) Cav-1 expression detected by western blot in cells transfected with Cav-1 or control (Ctl) siRNA. α-tubulin was used as loading control. Data are means ± SEM from 8 experiments. Control was taken as 100% and data are presented as the percentage changes relative to control conditions. *P<0.05 vs Ctl siRNA.

Supplementary figure 1

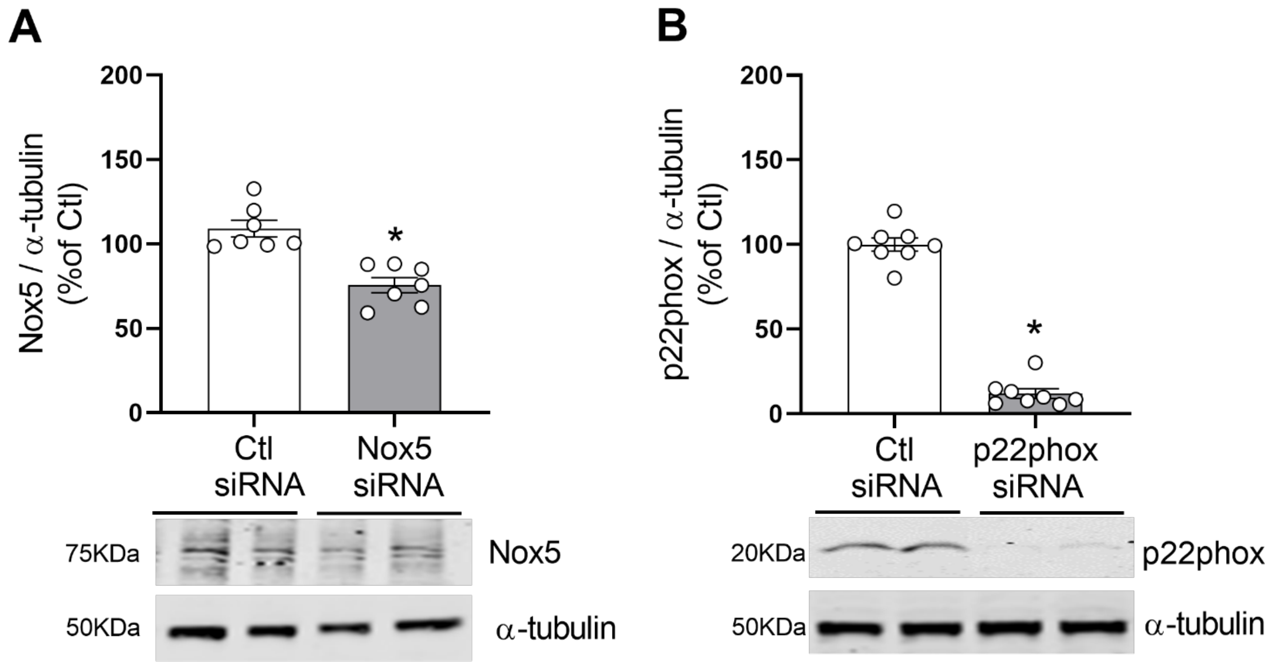

Supplementary figure 2

A

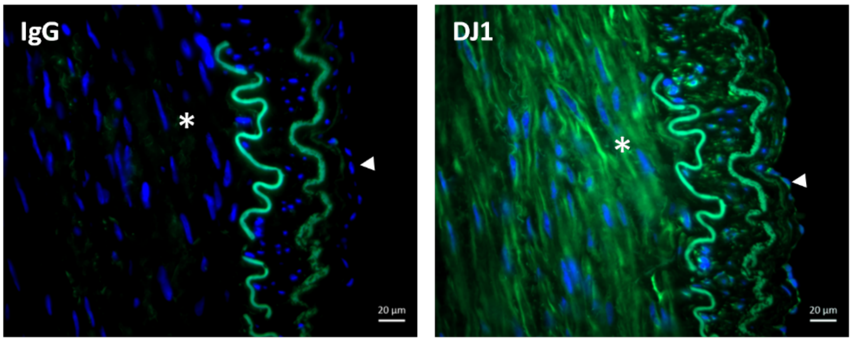

B

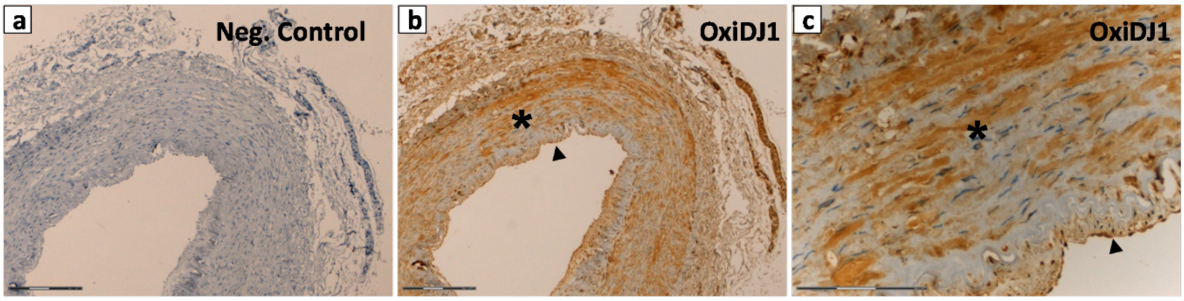

Supplementary figure 3

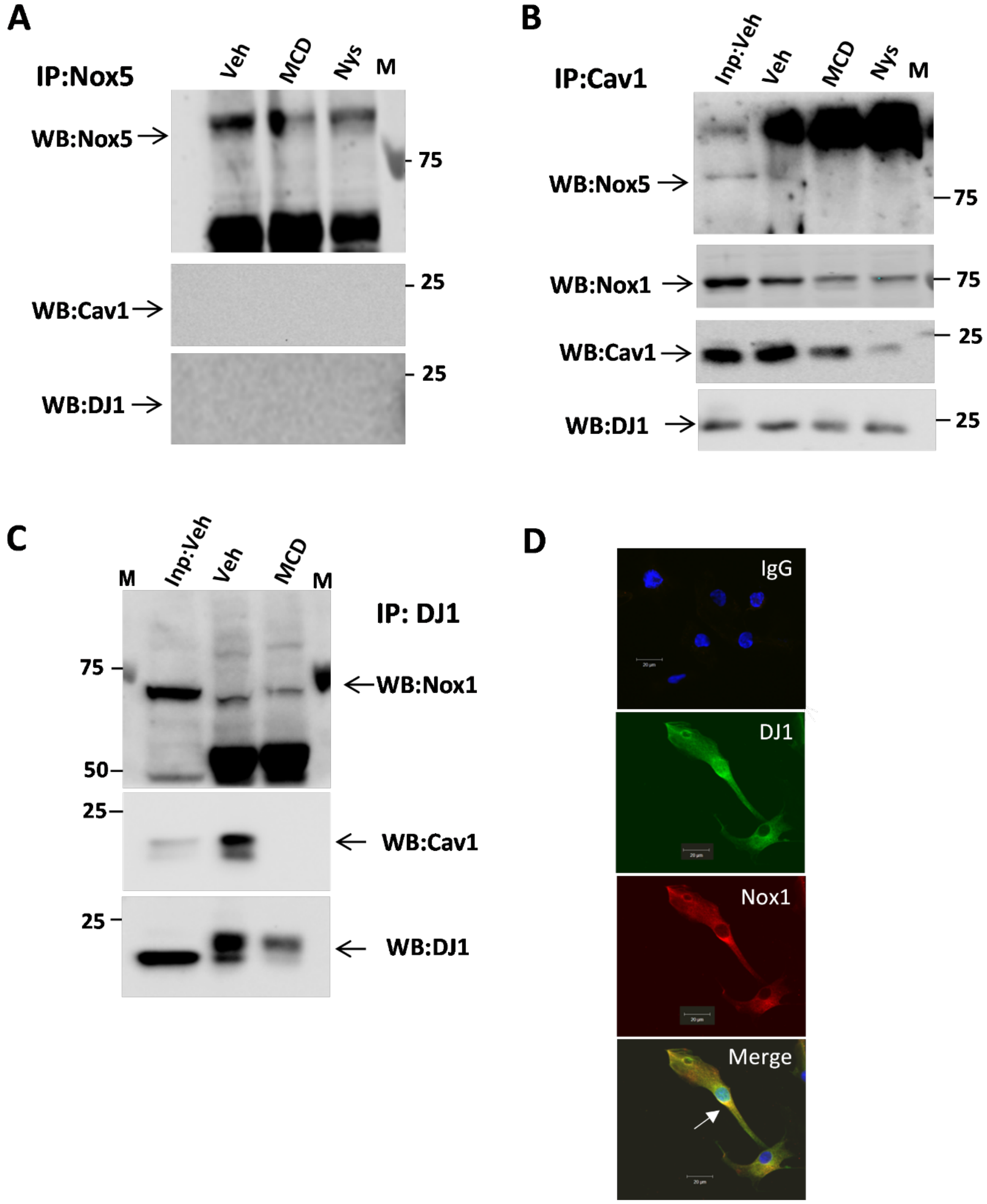

Supplementary figure 4

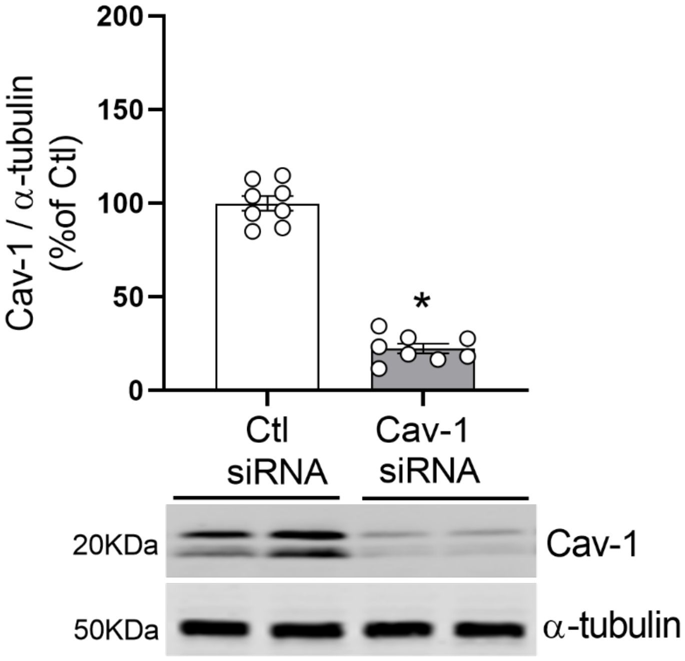

Supplement: Supplementary file 1 — Supplementary Information. [file 41598_2020_73751_MOESM1_ESM.pdf]
